# Association between Ulcerative Colitis, mental health, and Quality of Life in a Pakistani cohort: A cross-sectional analysis

**DOI:** 10.1371/journal.pone.0356531

**Published:** 2026-08-21

**Authors:** Saima Mushtaq, Sehrish Saeed, Amjad Khan, Tayyab Saeed Akhtar, Sameen Abbas, Bilal Ashraf, Yalin Dong, Weiyi Feng, Yu Fang

**Affiliations:** 1 Department of Pharmacy, The First Affiliated Hospital of Xi’an Jiaotong University, Xi’an, China; 2 Department of Pharmacy Administration and Clinical Pharmacy, School of Pharmacy, Health Science Center, Xi’an Jiaotong University, Xi’an, China; 3 Department of Pharmacy, Quaid-i-Azam University, Islamabad, Pakistan; 4 Center for Liver and Digestive Diseases, Holy Family Hospital, Rawalpindi, Pakistan; 5 Department of Anatomy, Islamabad Medical & Dental College, Islamabad, Pakistan; Universiti Teknologi MARA, MALAYSIA

## Abstract

**Background:**

Ulcerative colitis (UC) is chronic inflammatory bowel disease that significantly impairs both physical and psychological well-being. This study explores the prevalence of depression and its impact on quality-of-life (QoL) among UC patients in Pakistan, aiming to inform integrated care approaches in a setting where mental health often remains under-addressed.

**Methodology:**

A cross-sectional study was conducted at outpatient IBD clinics of Gastroenterology department of Holy Family Hospital, Rawalpindi, from February 2024 to May 2025. Data were collected using structured forms covering demographics, clinical history, and disease severity (assessed via Mayo Score and Montreal classification). Psychological status was evaluated using HADS and BDI-II, while QoL was assessed using the IBDQ-32.

**Result:**

Among 101 UC patients, anxiety was present in 21.8% and depression in 17.8% patients. The mean IBDQ score was 150 ± 32.9, with 12.9% reporting poor QoL, 60.4% good QoL, and 1% excellent QoL. Disease severity showed significant positive correlations with anxiety (ρ = 0.42,p = 0.001) and depression (ρ = 0.38,p = 0.002), and strong negative correlation with QoL (ρ = −0.51,p < 0.001). Kruskal–Wallis tests confirmed significant differences in psychological distress and QoL across severity groups (p < 0.05). Multivariable regression identified Mayo score, HADS-A, HADS-D, and disease extent as significant negative predictors of IBDQ score (p < 0.05).

**Conclusion:**

This study underscores strong correlation between higher Mayo scores, together with elevated levels of anxiety and depression, and poorer QoL as measured by IBDQ among patients with UC in Pakistan. Therefore, there is an important consideration to explore the psychological burden and QoL impairments faced by UC patients in Pakistan to inform holistic management strategies.

## Introduction

Ulcerative Colitis (UC), categorized under Inflammatory Bowel Disease (IBD), is a chronic, relapsing-remitting condition characterized by recurrent, uncontrolled inflammation of the large intestine, starting in the rectum and typically progressing proximally through varying segments, potentially affecting the entire colon in a continuous manner [1]. Symptoms may include abdominal pain, diarrhea, rectal bleeding, exhaustion, fever, and weight loss. The majority of UC patients experience cycles of worsened symptoms followed by a remission period [2,3]. Its etiology is multifactorial, involving genetic susceptibility, environmental triggers, gut microbiota disturbances, and immune dysregulation [4]. UC significantly impairs physical health, but its psychological burden is equally profound, often overlooked, and under-addressed in clinical practice, especially in low- and middle-income countries like Pakistan.

Globally, there is increasing recognition of the bidirectional relationship between chronic inflammatory conditions and mental health. Depression, in particular, is highly prevalent among individuals with UC, with reported rates ranging from 15% to 40% depending on disease activity, duration, and socio-demographic context [5,6]. The persistent and unpredictable nature of UC, the side effects of long-term medication use, and social stigma associated with gastrointestinal symptoms may exacerbate feelings of helplessness, anxiety, and depressive symptoms [3]. This psychological distress can, in turn, negatively influence disease outcomes by impairing adherence to therapy, reducing engagement in self-care, and triggering disease flares via the gut-brain axis.

Quality of life (QoL) is a multidimensional concept encompassing physical functioning, psychological state, social relationships, and overall perception of well-being [7]. In patients with UC, QoL is often diminished not only due to physical discomfort and disease limitations but also due to the emotional toll of living with a chronic condition. Research in high-income countries has demonstrated a clear link between disease activity, mental health, and diminished QoL in UC patients [8]. However, data from low-resource settings, such as Pakistan, remain limited. Cultural factors, unaffordability or unavailability of medications, lack of diagnostic facilities, social stigma, and lack of mental health awareness resulting in non-compliant behaviors because of a lack of understanding regarding the disease and its primary treatment may further exacerbate these challenges in the Pakistani context.

In Pakistan, the prevalence of UC appears to be increasing [9], potentially due to lifestyle changes, urbanization, and dietary shifts. In addition, advances in diagnostic capabilities may have improved case detection, thereby enhancing the accuracy of prevalence estimates. Despite this, the psychological dimensions of the disease have received minimal attention in both research and clinical settings. Most gastroenterology services focus on pharmacological disease control, often ignoring the mental health aspects that may significantly influence long-term outcomes. Given the scarcity of local data with no similar studies conducted in Pakistan, the aim was to assess anxiety and depressive symptoms along with their correlation with QOL in Pakistani UC patients.

## Methodology

### Study design and setting

A cross-sectional observational study was conducted at the outpatient IBD clinics of the Gastroenterology Department at Holy Family Hospital, Rawalpindi, from 1^st^ February 2024–15^th^ May 2025. The study received ethical approval from the Institutional Research and Ethics Forum of Holy Family Hospital, Rawalpindi, with protocol approval number 851/IREF/RMU/2024, and the Institutional Ethical Review Board and Bio-Ethical Committee (BEC) of Quaid-I-Azam University, Islamabad, with protocol approval number BEC-FBS-QAU2023-574.

### Study population and subjects

Convenience sampling was used to recruit participants based on their willingness and availability. The inclusion criteria for this study were as follows: being a resident of Pakistan, aged 18 years or older, with a confirmed diagnosis of UC based on clinical presentation, colonoscopic and histopathological evaluation, and consent to participate in the research. To ensure consistency with the data collection form, patients were required to present with at least one of the following documented symptoms: increased stool frequency, rectal bleeding, abdominal pain, or systemic symptoms such as weight loss or fatigue. Both newly diagnosed patients and those attending follow-up visits were enrolled.

### Study population and subjects

Exclusion criteria included patients admitted to the intensive care unit or critically unwell due to conditions unrelated to UC, who had not undergone a colonoscopy, those with Crohn's disease, previously diagnosed psychiatric disorders, and those taking anxiolytics and antidepressants; cognitive impairment preventing questionnaire completion; and incomplete clinical or questionnaire data. All data used in this study were entered and stored without any individual identifiers, such as full names, citizen ID numbers, or contact information. The data were used anonymously and did not reveal participants’ identities. The study was performed in accordance with the ethical principles of the Declaration of Helsinki (1975), as revised in 2013.

### Data collection method

A structured data collection form (S1 Table) was developed to obtain comprehensive information from participants. The form consisted of three main sections.

- The first section captured demographic details, including age, gender, weight, ethnicity, and socioeconomic status. This was followed by taking clinical history and presentation, including laboratory findings, treatment regimens, and assessment of disease severity using the Mayo Score at the time of visit, which evaluates four clinical and endoscopic parameters: stool frequency, mucosal appearance during endoscopy, rectal bleeding, and the physician’s global assessment, each scored from 0 to 3. A total score of ≤2 indicated remission, 3–5 was categorized as mild disease, 6–10 as moderate disease, and >10 as severe disease (Table 1).
- The second part was to detect the extent of disease and was categorized according to the Montreal classification: patients with inflammation limited to the rectum (distal to the rectosigmoid junction) were classified as having ulcerative proctitis (E1); those with inflammation up to the splenic flexure were labeled as having left-sided or distal colitis (E2); and patients with disease extending beyond the splenic flexure were categorized as having extensive colitis (E3).
- The third and most critical section of the form included validated tools to assess psychological health and QoL. Anxiety and depression were initially screened using the Hospital Anxiety and Depression Scale (HADS), while the Beck Depression Inventory-II (BDI-II) was used to determine the severity of depressive symptoms. QoL was evaluated using the disease-specific Inflammatory Bowel Disease Questionnaire (IBDQ-32).
  1. HADS is a 14-item questionnaire comprising two subscales: HADS-A (anxiety) and HADS-D (depression), with each subscale containing seven items. Each item is rated on a 4-point Likert scale ranging from 0 to 3, yielding a total score range of 0–21 per subscale. Based on standardized thresholds, scores were categorized as follows: 0–7 (normal), 8–10 (borderline), and ≥11 (abnormal). The already Urdu-validated version [10] was used in this study; Cronbach’s alpha was calculated, yielding values of 0.86 for HADS-A and 0.87 for HADS-D, indicating good internal consistency.
  2. BDI-II consists of 21 items, each rated on a 4-point scale (0–3), with a total score range of 0–63. BDI-II scores were categorized as follows: 0–13 (minimal), 14–19 (mild), 20–28 (moderate), and 29–63 (severe depression). The internal consistency of BDI-II (Urdu version [11]) was assessed using Cronbach’s alpha, which yielded a value of 0.924, indicating excellent reliability.
  3. Bowel, systemic, emotional, and social symptoms are the four domains that make up the IBDQ-32. Better HRQoL is marked by a higher total score, which ranges from 0 to 224. The instrument was used with permission from the concerned author [12].

**Table 1:** Components of the Mayo Score used for disease severity.

Score	Criteria
Stool Frequency	
0	Normal
1	1-2 stools/day more than normal
2	3-4 stools/day more than normal
3	5 or more stools/day more than normal
Rectal Bleeding	
0	None
1	Streaks of blood with stool less than half the time
2	Obvious blood with stool most of the time
3	Blood alone passed
Mucosal Appearance during Colonoscopy	
0	Normal
1	Mild disease (erythema, decreased vascular pattern, mild friability)
2	Moderate disease (marked erythema, absent vascular pattern, friability, erosions)
3	Severe disease (spontaneous bleeding, ulceration)
Physician Global Assessment	
0	Normal
1	Mild disease
2	Moderate disease
3	Severe disease
Interpretation	Normal/Remission: 0–2 Mild disease activity: 3–5 Moderate disease activity: 6–10 Severe disease activity: > 10

Data were collected during patient visits, where face-to-face interviews were conducted to obtain clinical information, evaluate risk factors, and record past medical history. All participants provided informed consent prior to their inclusion.

### Inclusivity in global research

Additional information regarding the ethical, cultural, and scientific considerations specific to inclusivity in global research is included in the Supporting Information (S2 Table).

### Statistical analysis

Data were analyzed using IBM SPSS Statistics, version 25 (SPSS Inc., Chicago, IL). Missing data were excluded from the final analysis and were not imputed. Descriptive statistics were used to summarize demographic and clinical characteristics. For anxiety and depression severity, participants were classified according to the standard validated cut-off values of the HADS and BDI-II instruments. For selected statistical analyses (i.e., chi-square tests for categorical variables and t-tests or ANOVA for continuous variables), these severity categories were numerically coded (e.g., normal/minimal, borderline/mild, abnormal/moderate-severe) to facilitate group comparisons and regression analyses. Accordingly, the reported mean values represent the coded category scores rather than the original cumulative questionnaire scores. Classification into severity groups was based on the standard validated cut-off values of each instrument.

A correlation between the scales and the Mayo score was determined using Spearman’s correlation. Multicollinearity diagnostics were assessed during regression modeling, and no significant multicollinearity concerns were identified among the included variables, including HADS-D and BDI-II. Basic model assumption checks, including assessment of residual distribution and homoscedasticity, were also reviewed to ensure the appropriateness of the linear regression model. A p-value of <0.05 was considered statistically significant.

## Results

All patients who met the study's inclusion criteria were included using a convenience sampling technique. A total of 155 individuals visited the IBD clinic and were enrolled. Duplicate records were removed, and the final sample size was 101 participants after applying exclusion criteria and excluding participants with missing or insufficient data. The mean age of the participants was 35.93 ± 12.00 years, and the mean weight was 59.05 ± 11.15 kg. The majority of the study population was male, = 57 individuals (56.4%), while females accounted for 44 (43.6%). The overall mean hemoglobin level was 11.08 ± 6.38 g/dL, with males exhibiting significantly higher levels (12.30 ± 8.20 g/dL) than females (9.61 ± 2.11 g/dL). This difference was statistically significant (mean difference = 2.69 g/dL; 95% CI: 0.05 to 5.344; p = 0.046). In contrast, no significant difference was observed in erythrocyte sedimentation rate (ESR) values between males and females. The overall mean ESR was 37 ± 19.95 mm/hr, with males and females having similar means (34.95 ± 17.78 mm/hr vs. 38.10 ± 20.60 mm/hr, respectively), and the difference was not statistically significant (p = 0.438). The average white blood cell count was 9036 ± 360 per mm³, and the platelet count was 337,000 ± 110 per mm³. The average hematocrit was recorded as 33.04 ± 7.70% (Table 2).

**Table 2:** Baseline characteristics of patients with UC (n = 101).

Category	Variable	Mean ± SD / Frequency (%)	P-Value
Demographics	Age (years)	35.93 ± 12.00	
	Weight (kg)	59.05 ± 11.15	
	Male	57 (56.4)	
	Female	44 (43.6)	
Presenting Complaints	Blood in stool	64 (69.6)	
	Diarrhea	75 (81.5)	
	Abdominal pain	81 (88)	
	Mucus in stool	32 (34.8)	
	Pain with defecation	70 (76.1)	
	Weight loss	39 (42.4)	
Laboratory Parameters	Hemoglobin (g/dL)	11.08 ± 6.38	0.046*
	RBC count (million/mm³)	4.33 ± 0.94	0.127
	WBC count (/mm³)	9,036 ± 360	0.310
	Platelet count (/mm³)	337,000 ± 110	0.169
	ESR (mm/hr)	37 ± 19.95	0.438
	Hematocrit (%)	33.04 ± 7.70	0.001*
(Mayo Score)	Normal or Inactive Disease	04 (04)	
	Mild (3–5)	15 (14.9)	
	Moderate (6–10)	40 (39.6)	
	Severe (> 10)	42 (41.6)	
Disease Extent (Montreal Classification)	E1: Ulcerative proctitis	22 (21.8)	
	E2: Left-sided colitis	31 (30.7)	
	E3: Extensive colitis	48 (47.5)	
Histopathological findings	Cryptitis	87 (94.6)	
	Distortion of crypt architecture	60 (65.2)	
	Crypt abscess	55 (59.8)	
	Mucin-depleted goblet cells	53 (57.6)	
	Dysplasia with intact basement membrane	8 (8.7)	
	Dysplasia with invasion of the basement membrane (malignancy)	0 (0)	

*Statistically significant at p < 0.05, SD Standard Deviation, RBC Red Blood Cell, WBC White Blood Cell, ESR Erythrocyte Sedimentation Rate.

**Fig 1:**
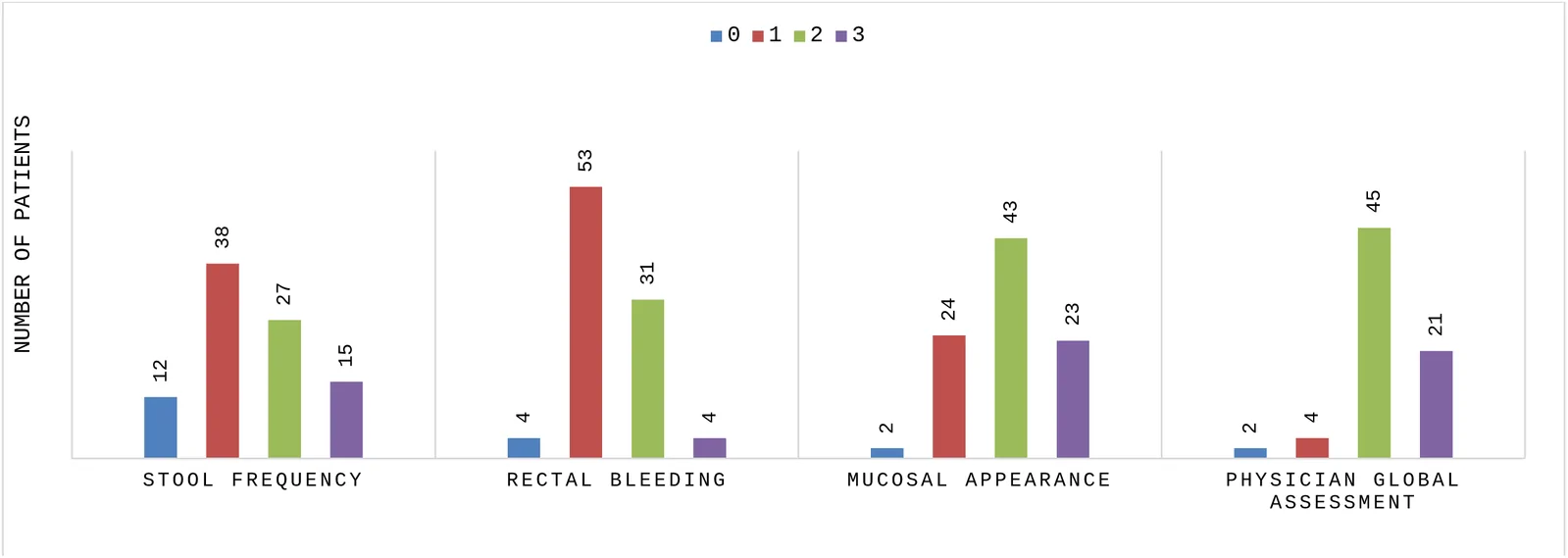
Components of the Mayo Score and the corresponding number of patients in each subcategory.

Disease severity was assessed using the Mayo Score. This validated clinical index categorizes UC into four levels: remission (inactive disease), mild, moderate, and severe. In this study, 4% of patients were in clinical remission, 14.9% had mild disease, 39.6% had moderate disease, and 41.6% had severe UC. A detailed breakdown of each component of the Mayo Score, along with the corresponding number of patients in each subcategory, is illustrated in Fig 1 and Table 2.

Disease extent was defined using the Montreal classification as previously described. Patients were assigned to one of the three categories (E1, E2, or E3) based on disease extent. There was a fairly equal distribution of disease among these three categories, with 21.8% (n = 22) in the E1 (ulcerative proctitis), 30.7% (n = 31) in the E2 (left-sided colitis), and 47.5% (n = 48) in the E3 (extensive colitis) group. This was further confirmed by the histopathological findings in colonic biopsy samples of patients diagnosed with UC. The most frequently identified feature was cryptitis, present in 94.6% of patients, followed by distortion of crypt architecture (65.2%), crypt abscesses (59.8%), and mucin-depleted goblet cells (57.6%). Dysplasia with intact basement membrane was observed in 8.7% of cases, while no cases of invasive dysplasia or malignancy were identified (Table 2).

Among the 101 patients assessed, the mean anxiety score (HADS-A) was 1.68 ± 0.812. Based on the scoring system, 53.5% (n = 54) of patients were categorized as normal, 24.8% (n = 25) as borderline, and 21.8% (n = 22) as abnormal, indicating clinically significant anxiety symptoms. For the HADS-D subscale, the mean score was 1.49 ± 0.782. Of the participants, 68.3% (n = 68) were classified as normal, 13.9% (n = 14) were borderline, and 17.8% (n = 18) were categorized as having depression. Based on the BDI-II, the mean depression score was 1.48 ± 0.912. The distribution of depression severity is shown in Table 3.

**Table 3:** Characteristics of HADS-A, HADS-D, and BDI-II scores in UC patients (n = 101).

Characteristics	N (%)	Mean ± SD
**Total Anxiety score**		
Normal	54 (53.5%)	1.68 ± 0.812
Borderline-Abnormal	25 (24.8%)	
Abnormal	22 (21.8%)	
**Total Depression Score**		
Normal	69 (68.3%)	1.49 ± 0.782
Borderline-Abnormal	14 (13.9%)	
Abnormal	18 (17.8%)	
**Beck Depression Inventory Score**		
None or minimal depression	75 (74.3%)	1.48 ± 0.912
Mild depression	09 (8.9%)	
Moderate depression	11 (10.9%)	
Severe depression	06 (5.9%)	

**Fig 2:**
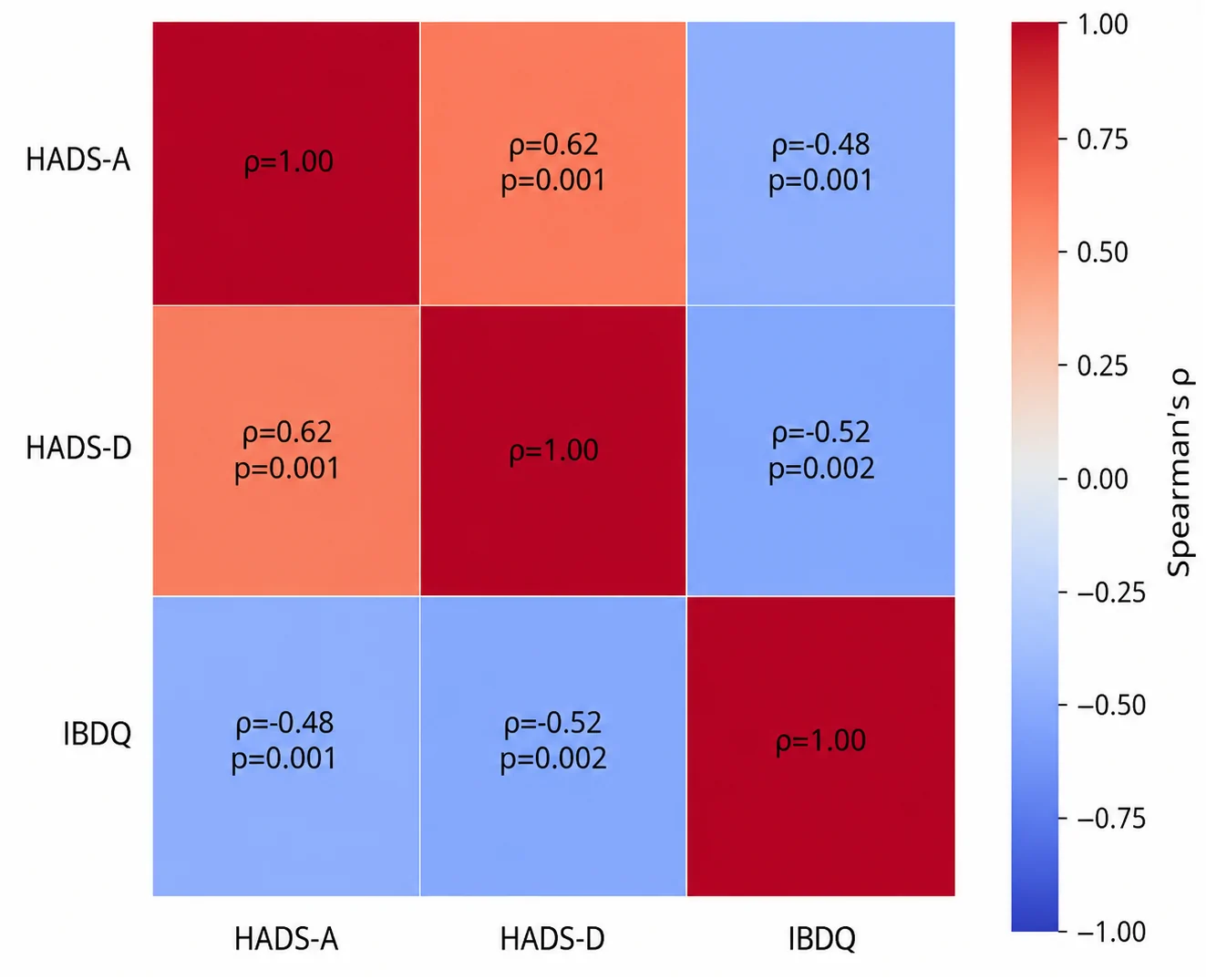
Heatmap showing correlation between anxiety, depression, and QoL (blue = positive correlation, red = negative). Significant positive correlations were observed between Mayo Score and both HADS-A (ρ = 0.42, p = 0.001) and HADS-D (ρ = 0.38, p = 0.002), suggesting that disease severity was associated with increased anxiety and depression. In contrast, Mayo Score was negatively correlated with IBDQ total score (ρ = −0.51, p < 0.001), indicating a strong inverse relationship between disease severity and QoL. Furthermore, HADS-A and HADS-D were strongly positively correlated (ρ = 0.62, p < 0.001), and both were significantly negatively correlated with IBDQ (ρ = −0.58 and −0.61, respectively; p < 0.001), reinforcing the impact of psychological distress on QoL (Table 4).

Based on IBDQ score classification thresholds, 12.9% of patients had a low QoL, 25.7% had a regular QoL, 60.4% had a good QoL, and only 1% had an excellent QoL. The mean scores for the four domains of the IBDQ-32 were as follows: bowel symptoms (44.7 ± 10.11), systemic symptoms (20.8 ± 4.95), emotional function (58.2 ± 13.14), and social function (26.1 ± 6.36). Overall, the IBDQ score had a mean of 150 ± 32.9, reflecting moderate impairment in QoL among the study population. A correlation heatmap showing the correlations between anxiety (HADS-A), depression (HADS-D), and QoL (IBDQ) is represented in Fig 2 showing anxiety and depression were positively correlated (ρ = 0.62, p < 0.01), while both showed significant negative correlations with QoL (HADS-A: ρ = –0.48, p < 0.01; HADS-D: ρ = –0.52, p < 0.01) (Table 4).

**Table 4:** Spearman's correlation between disease severity (Mayo score), psychological distress (HADS), and QoL (IBDQ) in UC patients (n = 101).

Variables	Mayo Score	HADS-A (Anxiety)	HADS-D (Depression)	IBDQ Total Score
**Mayo Score**	1.00	ρ = 0.42, *p* = 0.001*	ρ = 0.38, *p* = 0.002*	ρ = -0.51, *p* < 0.001*
**HADS-A (Anxiety)**		1.00	ρ = 0.62, *p* < 0.001*	ρ = -0.48, *p* < 0.001*
**HADS-D (Depression)**			1.00	ρ = -0.52, *p* = 0.002*
**IBDQ Total Score**				1.00

*Statistically significant at p < 0.05; ρ: Spearman’s rho; HADS: Hospital Anxiety and Depression Scale; IBDQ: Inflammatory Bowel Disease Questionnaire.

Univariate regression analyses demonstrated that higher Mayo scores, extensive disease extent, and elevated anxiety, depression, and BDI-II scores were each significantly associated with lower IBDQ QoL scores (all p < 0.01). In contrast, age, sex, and weight did not show significant associations. A multivariable linear regression was conducted to identify predictors of QoL (measured by total IBDQ score) among patients with UC, with approximately 56% of the variance in QoL (Adjusted R² = 0.56). The model showed that the Mayo Score was a strong negative predictor of QoL (B = −5.78, p = 0.01), indicating that increased disease severity was associated with lower IBDQ scores. Similarly, anxiety (B = −2.91, p = 0.008) and depression scores (B = −3.84, p < 0.001) were significantly associated with reduced QoL. Regarding disease extent, patients with extensive colitis (E3) had significantly lower IBDQ scores compared to those with ulcerative proctitis (B = −9.27, p = 0.023). Demographic variables, including age and gender, did not demonstrate significant associations in the adjusted model (Table 5).

**Table 5:** Univariate and multivariable linear regression analysis identifying predictors of QoL (IBDQ total score) in UC patients (n = 101).

Predictor Variable	Univariate β (95% CI)	P-value	Multivariable β (95% CI)	P-value
Age (years)	−0.12 (−0.34 to 0.10)	0.285	−0.08 (−0.28 to 0.12)	0.186
Sex (Male vs. Female)	1.85 (−4.26 to 7.96)	0.552	0.92 (−3.95 to 5.79)	0.408
Weight (kg)	0.08 (−0.15 to 0.31)	0.490	0.05 (−0.16 to 0.26)	0.640
Mayo Score	−4.85 (−6.92 to −2.78)	<0.001*	−3.94 (−6.12 to −1.76)	0.01*
Disease Extent (E3 vs. E1)	−6.24 (−10.8 to −1.68)	0.008*	−4.12 (−8.05 to −0.19)	0.023*
HADS-Anxiety Score	−3.92 (−5.87 to −1.97)	<0.001*	−2.46 (−4.50 to −0.42)	0.018*
HADS-Depression Score	−4.45 (−6.44 to −2.46)	<0.001*	−2.88 (−5.05 to −0.71)	0.01*
BDI-II Score	−5.12 (−7.18 to −3.06)	0.01*	−3.36 (−5.49 to −1.23)	0.002*

*Statistically significant at p < 0.05; HADS: Hospital Anxiety and Depression Scale; BDI-II: Beck Depression Inventory, β: Standardized coefficient.

## Discussion

This study evaluated the relationship between disease activity, psychological distress, and HRQoL among patients with UC in Pakistan. The findings demonstrated that greater disease severity, as assessed by the Mayo score, was significantly associated with higher levels of anxiety and depression and poorer HRQoL. Furthermore, multivariable regression analysis identified disease severity, anxiety, depression, and extensive disease extent as independent predictors of reduced QoL. These findings highlight the substantial psychological burden associated with ulcerative colitis and emphasize the close interplay between clinical disease activity and patient-reported outcomes.

Our findings are consistent with previous studies demonstrating that active UC is closely associated with increased psychological distress and impaired QoL [13,14]. UC is a chronic condition often presented with a significant diagnostic delay, leading to late identification in many patients who may initially be unaware of the chronic and progressive nature of their condition. This late diagnosis often means that patients have already experienced prolonged periods of untreated symptoms, which can contribute to disease progression and greater severity, most commonly in countries like Pakistan [15]. Once diagnosed, UC necessitates lifelong treatment, and adherence to pharmacotherapy is paramount for achieving optimal outcomes. However, non-adherence is a common challenge, leading to late diagnosis and subsequent non-adherence, which significantly increases the risk of symptomatic relapses, colorectal cancer, and escalating healthcare costs [1,2].

Individuals with chronic illnesses, including IBD, face a heightened susceptibility to anxiety and depression compared to the general population, consequently diminishing their QoL due to the mentioned factors. Specifically, patients with active IBD demonstrate a higher prevalence of anxiety and depressive symptoms than those in remission. Our study aimed to address this gap in the local literature by assessing the prevalence of depressive symptoms and evaluating QoL among UC patients in Pakistan using validated tools, with the aim of informing targeted mental health interventions and integrated care models.

The prevalence of depression and anxiety in UC patients varies across studies. Previous research reported prevalence rates of 25.2% for depression and 32.1% for anxiety in UC patients, with these rates rising to 38.9% and 57.6%, respectively, in patients with active disease [16]. A Brazilian study identified even higher rates, with 65.52% of UC patients experiencing anxiety and 28.87% experiencing depression [12]. In contrast, an Israeli study on IBD patients found lower prevalences of 9.36% for depression and 23.4% for anxiety [17]. Our study, involving 101 UC patients, found that 46.6% experienced anxiety or were at the borderline stage, while 31.7% had depression or were at the borderline level. Differences in sampling techniques, assessment tools, and inclusion criteria may account for variations observed across studies.

The most likely explanation for these results is that psychological responses to the social, cultural, and physical difficulties associated with IBD frequently result in anxiety and depression. Chronic diarrhea, abdominal discomfort, weakness, rectal bleeding, and general weakness are some of the symptoms of UC that make it difficult for the patient to engage in everyday social and professional activities. Adverse drug reactions might also exacerbate depressive symptoms. For example, corticosteroids, which are frequently used to treat IBD, have a substantial correlation with mental health issues, such as the exacerbation of depressive symptoms. IBD patients have psychological discomfort due to a variety of additional burdens, including difficulties in relationships, clinical issues, and social stigmatization, in addition to physical symptoms and side effects from medication [18], especially in Pakistan. Social expectations, family honor, and reliance on faith-based or traditional remedies may delay formal psychiatric consultation. These cultural and systemic barriers contribute to the underestimation of psychological burden in UC patients and highlight the urgent need for culturally sensitive screening and integrated care strategies.

Patient’s overall health status and daily functioning following treatment are determined by HRQoL, providing insights into clinical endpoints and therapeutic efficacy [19]. While general QoL tools like EuroQol (EQ)-5D and the Medical Outcomes Study 36-Item Short Form (SF-36) exist, IBDQ-32’s specific design for IBD patients makes it more suitable for comprehensive assessment. Our study’s mean QoL score (150) was consistent with that reported in a study conducted in Iran (156.9), despite differences in sample size and gender distribution [20].

Consistent with prior research, our study found that HRQoL was lower during periods of active disease, further supporting the notion that disease severity significantly impacts the QoL in UC patients [21]. Our findings align with previously reported results, reinforcing the correlation between anxiety, depression, and decreased QoL, with a negative correlation in IBD patients. Notably, only a small percentage of individuals in our study (1%) reported an excellent QoL, which is consistent with other studies where only 3.6% (n = 110) and 7% (n = 97) of participants achieved high QoL scores [12,21]. Patients requiring hospitalization for UC-related complications, those with anemia, and individuals experiencing poor sleep quality also report lower HRQoL [20]. These consistent findings across multiple studies underscore the critical need for comprehensive management strategies in IBD that address both physical symptoms and mental health, with effective mental health interventions, such as counseling, stress management, and targeted pharmacological treatments, being crucial for improving patients’ overall well-being.

This study has several notable strengths. First, it is the first to comprehensively assess psychological distress and QoL in UC patients in Pakistan, integrating validated scales (HADS, BDI-II, and IBDQ-32) with disease severity (Mayo score) and extent (Montreal classification). Second, by combining both clinical and patient-reported outcomes, the study provides a multidimensional perspective on the disease burden, bridging a critical evidence gap in low-resource settings. Finally, the inclusion of robust statistical analyses, including correlation and regression modeling, strengthens the validity of the findings and provides a foundation for future longitudinal and interventional research.

As the study had a cross-sectional design, it limited the ability to infer causality among disease activity, detailed information regarding participants’ treatment regimens (including medication type, duration of therapy, biologic use, and corticosteroid exposure), psychological distress, and QoL, which was a fundamental limitation. Longitudinal studies with multicenter and community-based cohorts with larger sample sizes and detailed treatment data are warranted to better understand the relationship between therapeutic interventions and psychological outcomes. Second, the use of convenience sampling from a single tertiary care center with smaller sample size may limit the generalizability of findings to the broader Pakistani UC population, particularly those in rural or under-resourced settings. Third, although validated tools were used to minimize comprehension barriers, each questionnaire was administered by the primary investigator, who explained items in Urdu when necessary. To overcome these, intervention-based trials that integrate psychosocial support, psychotherapy, or psychiatric consultation into routine IBD care could provide valuable insight into improving overall outcomes.

## Conclusion

This study demonstrates significant associations between disease severity, psychological distress, and reduced QoL among UC patients in Pakistan, with higher Mayo scores correlating with elevated anxiety, depression, and poorer IBDQ scores. While these findings emphasize the psychological burden in UC, they should be interpreted within the limitations of a cross-sectional design and single-center sample. Beyond our cohort, the results support the importance of incorporating routine psychological screening and culturally sensitive mental health support into IBD management, particularly in low- and middle-income countries, where stigma and under-recognition of mental health remain major challenges.

## Supporting information

S1 Table Data collection form. (DOCX)

S2 Table Inclusivity in global research. (DOCX)

## Abbreviations

UC: Ulcerative Colitis
IBD: Inflammatory Bowel Disease
QoL: Quality of Life
HADS: Hospital Anxiety and Depression Scale
BDI-II: Beck Depression Inventory-II
IBDQ-32: Disease-specific Inflammatory Bowel Disease Questionnaire
ESR: Erythrocyte sedimentation rate
NSAIDs: Non-steroidal anti-inflammatory drugs
