# Supplementary material for: Association between Ulcerative Colitis, mental health, and Quality of Life in a Pakistani cohort: A cross-sectional analysis
Source: PLoS One. 2026 Aug 21;21(8):e0356531. doi: 10.1371/journal.pone.0356531 (PMC13496436; doi:10.1371/journal.pone.0356531)
Supplement: Supplementary file 1 [file pone.0356531.s001.docx]

**Data Collection Form**

A). **Demographics/ History/ Diagnosis**

| Registration No |  | Gender | | - Male | | - Female |
| --- | --- | --- | --- | --- | --- | --- |
| Age (Years) |  | Age group | | - Child ○ Adult ○ Old | | |
| Weight (kg) |  | Height (cm) | |  | | |
| BMI |  | Resident | | - Rural ○ Urban | | |
| Socio-Economic Status | - Low ○ Middle ○ High | | | | | |
| Education Level | - No formal Education ○ Primary ○ Secondary ○ Tertiary ○   Unclassified | | | | | |
| Address (District) |  | | | | | |
| Marital Status | - Single ○Married ○ Widow - Divorced | | | | | |
| Race | - Punjabi ○ Pathan ○ AJK ○Sindhi Others: | | | |  |  |
| Smoking Status | - Ever Smoker ○ Current Smoker ○ Ex-Smoker   - Non-Smoker | | | | | |
| Alcohol | - Current drinker ○ Ex-drinker ○ Non-drinker | | | | | |
| Drug Addiction | - Current DA ○ Ex-DA ○ No DA | | | | | |
| Employment | - Employed (○ Government, ○Private - Self Employed - Un-employed - Retired - House Wife - Student | | | | | |
| **Association with Risk group** ○ Yes ○No | | | | | | |
| - Chronic NSAIDs use ○ Chronic Oral contraceptive Use - Smoking history ○ Family history - Intestinal parasite exposure ○ Appendectomy | | | | | | |
| **DIAGNOSIS** | | | | | | |
| Stool frequency | ○ <4 ○ 4-6 ○  >6 | | Blood in stool | | - Mild ○   Moderate   - Severe | |
| Weight loss | ○ <5 ○ 5-10   - >10 | | Abdominal pain | | - Yes - No | |
| General wellbeing |  | | Weight loss | |  | |

**Previous History of Surgical intervention**

Patients have previous history ○ Yes ○ No (If Yes, please state)

**Chronic use of NSAIDs** ○ Yes ○ No

**Use of anti-depressant** ○ Yes ○ No

B). **Clinical presentation of patients (on admission)**

Tick the option below

| **Symptoms** | |
| --- | --- |
| PR Bleeding | - Yes ○ No |
| Chronic diarrhea | - Yes ○ No |
| Blood in stools | - Yes ○ No |
| Abdominal pain | - Yes ○ No |
| Fever | - Yes ○ No |
| Anemia | - Yes ○ No |
| Anorexia | - Yes ○ No |
| Mucus in stools | - Yes ○ No |
| Pain during defecation | - Yes ○ No |
| Fatigue | - Yes ○ No |

C). **Laboratories Findings**

| WBC (×10^9^/L) |  | ESR |  |
| --- | --- | --- | --- |
| RBC (×10^12^/L) |  | CPR |  |
| PLT (×10^9^/L) |  |  |  |
| HGB (g/dL) |  |  |  |
| HCT (%) |  |  |  |

D). **Treatment Regimen**

| **S.No** | **Medicine prescribed** | **Route** | **Dosage** | **Dosing regimen** | **Specific Reason (if)** |
| --- | --- | --- | --- | --- | --- |
| **1** |  |  |  |  |  |
| **2** |  |  |  |  |  |
| **3** |  |  |  |  |  |
| **4** |  |  |  |  |  |

**Hospital Anxiety and Depression Scale (HADS)**

**Tick the box beside the reply that is closest to how you have been feeling in the past week. Don’t take too long over you replies: your immediate is best.**

**D**	A	**D**

###### I feel tense or 'wound up':

3	Most of the time	3

2	A lot of the time	2

1	From time to time, occasionally	1

0	Not at all	0

###### I still enjoy the things I used to enjoy:

0 Definitely as much
1 Not quite so much
2 Only a little
3 Hardly at all

###### I get a sort of frightened feeling as if something awful is about to happen:

3	Very definitely and quite badly	3

2	Yes, but not too badly	2

1	A little, but it doesn't worry me	1

0	Not at all	0

###### I can laugh and see the funny ide of things:

0 As much as I always could
1 Not quite so much now
2 Definitely not so much now
3 Not at all

###### Worrying thoughts go through my mind:

3	A great deal of the time	0

2	A lot of the time	1

1	From time to time, but not too	2

often

0	Only occasionally	3

###### I feel cheerful:

3	Not at all

2	Not often

1	Sometimes

0	Most of the time

###### I can sit at ease and feel relaxed:

**A**

###### I feel as if I am slowed down:

Nearly all the time Very often Sometimes

Not at all

###### I get a sort of frightened feeling ike 'butterflies' in the stomach:

0	Not at all

1	Occasionally

2	Quite Often

3	Very Often

###### I have lost interest in my appearance:

Definitely

I don't take as much care as I hould

I may not take quite as much care I take just as much care as ever

###### I feel restless as I have to be on he move:

3	Very much indeed

2	Quite a lot

1	Not very much

0	Not at all

###### I look forward with enjoyment o things:

As much as I ever did Rather less than I used to Definitely less than I used to

Hardly at all

###### I get sudden feelings of panic:

3	Very often indeed

2	Quite often

1	Not very often

0	Not at all

###### I can enjoy a good book or radio or TV program:

| 0 | Definitely | 0 |  | Often |
| --- | --- | --- | --- | --- |
| 1 | Usually  | 1 |  | Sometimes  |
| 2 | Not Often | 2 |  | Not often |
| 3 | Not at all | 3 |  | Very seldom |

**Beck Depression Inventory II (BDI-II)**

This depression inventory can be self-scored. Please read each group of statements carefully. And then pick out the one statement in each group that best describes the way you have been feeling during the past two weeks, including today. The scoring scale is at the end of the questionnaire. Rate your feelings from 0-3

0=not at all, 1=occasionally ,2=a lot of time,3=Most of time

| **1** | Sadness | 0  (not at all) | 1  (sometime) | 2  (A lot of time) | 3  (Most of time) |
| --- | --- | --- | --- | --- | --- |
| **2** | Pessimism | 0 | 1 | 2 | 3 |
| **3** | Past failure  (feels like a failure) | 0 | 1 | 2 | 3 |
| **4** | Loss of pleasure | 0 | 1 | 2 | 3 |
| **5** | Guilty feeling | 0 | 1 | 2 | 3 |
| **6** | Punishment feeling | 0 | 1 | 2 | 3 |
| **7** | Self-dislike  (lost confidence) | 0 | 1 | 2 | 3 |
| **8** | Self-criticalness (criticize myself) | 0 | 1 | 2 | 3 |
| **9** | Suicidal thoughts or wishes | 0 | 1 | 2 | 3 |
| **10** | Crying | 0 | 1 | 2 | 3 |
| **11** | Agitation | 0 | 1 | 2 | 3 |
| **12** | Loss of interest | 0 | 1 | 2 | 3 |
| **13** | Indecisiveness | 0 | 1 | 2 | 3 |
| **14** | Worthlessness | 0 | 1 | 2 | 3 |
| **15** | Loss of energy | 0 | 1 | 2 | 3 |
| **16** | Changes in sleep pattern | 0 | 1 | 2 | 3 |
| **17** | Irritability | 0 | 1 | 2 | 3 |
| **18** | Changes in appetite | 0 | Somewhat less or greater | Much less or greater | No appetite at all |
| **19** | Concentration Difficulty | 0 | 1 | 2 | 3 |

| **20** | Tiredness or fatigue | 0 | 1 | 2 | 3 |
| --- | --- | --- | --- | --- | --- |
| **21** | Loss of interest in sex | 0 | 1 | 2 | 3 |

###### Inflammatory Bowel Disease Questionnaire (IBDQ-32)

1. How often have you had bowel movements in the last two weeks? Please indicate how often you have had a bowel movement in the last two weeks by choosing one of the following options

| Almost | Almost always | Often | Few times | Very few times | Rarely | Never |
| --- | --- | --- | --- | --- | --- | --- |

2. How often have you felt tired, fatigued and exhausted in the last two weeks?

| Almost | Almost always | Often | Few times | Very few times | Rarely | Never |
| --- | --- | --- | --- | --- | --- | --- |

3. How often in the last two weeks have you felt frustrated, impatient or restless?

| Almost | Almost always | Often | Few times | Very few times | Rarely | Never |
| --- | --- | --- | --- | --- | --- | --- |

4. How often in the last two weeks have you not been able to go to school or work because of your intestinal problem?

| Almost | Almost always | Often | Few times | Very few times | Rarely | Never |
| --- | --- | --- | --- | --- | --- | --- |

5. How often in the last two weeks have you had diarrhea?

| Almost | Almost always | Often | Few times | Very few times | Rarely | Never |
| --- | --- | --- | --- | --- | --- | --- |

6. How much physical fitness did you feel you had in the last two weeks?

| No power | Too  little power | Low energy | Some energy | Moderate  amount of energy | Plenty  of energy | Full of energy |
| --- | --- | --- | --- | --- | --- | --- |

7. How often in the last two weeks have you felt worried about the possibility of needing surgery because of your intestinal problem?

| Almost | Almost always | Often | Few times | Very few times | Rarely | Never |
| --- | --- | --- | --- | --- | --- | --- |

8. How often in the last two weeks have you had to delay or cancel a social engagement because of your intestinal problem?

| Almost | Almost always | Often | Few times | Very few times | Rarely | Never |
| --- | --- | --- | --- | --- | --- | --- |

9. How often in the last two weeks have you had stomach cramps?

| Almost | Almost always | Often | Few times | Very few times | Rarely | Never |
| --- | --- | --- | --- | --- | --- | --- |

10. How often in the last two weeks have you felt unwell?

| Almost | Almost always | Often | Few times | Very few times | Rarely | Never |
| --- | --- | --- | --- | --- | --- | --- |

11. How often in the last two weeks have you had problems because you were afraid of not finding a bathroom?

| Almost | Almost always | Often | Few times | Very few times | Rarely | Never |
| --- | --- | --- | --- | --- | --- | --- |

12. How much difficulty have you had in playing sports or having fun as you would like to have, because of your intestinal problems, in the last two weeks?

| Impossible to carryout activity | Great difficulty | Moderate activity | Some activity | Little activity | Rarely any difficulty | No difficulty |
| --- | --- | --- | --- | --- | --- | --- |

13. How often in the last two weeks have you been bothered by stomach pains?

| Almost | Almost always | Often | Few times | Very few times | Rarely | Never |
| --- | --- | --- | --- | --- | --- | --- |

14. How often in the last two weeks have you had problems getting a good night's sleep or waking up during the night? (due to intestinal problems)

| Almost | Almost always | Often | Few times | Very few times | Rarely | Never |
| --- | --- | --- | --- | --- | --- | --- |

15. How often in the last two weeks have you felt depressed and lacking courage?

| Almost | Almost always | Often | Few times | Very few times | Rarely | Never |
| --- | --- | --- | --- | --- | --- | --- |

16. How often, in the last two weeks, did you avoid going to places that did not have bathrooms (private) nearby?

| Almost | Almost always | Often | Few times | Very few times | Rarely | Never |
| --- | --- | --- | --- | --- | --- | --- |

17. In general, in the last two weeks, how much problem did you have with eliminating a large amount of gas?

| Main problem | Big problem | An imp problem | Any problem | Little problem | Rarely a problem | No problem |
| --- | --- | --- | --- | --- | --- | --- |

18. In general, in the last two weeks, how much trouble did you have in maintaining your weight as you would like it to be?

| Main problem | Big problem | An imp problem | Any problem | Little problem | Rarely a problem | No problem |
| --- | --- | --- | --- | --- | --- | --- |

19. Many patients with intestinal problems often have worries and anxiety about their illness. This includes worries about cancer, worries about never feeling better again, worries about getting worse. How often in the last two weeks have you felt worried and anxious?

| Almost | Almost always | Often | Few times | Very few times | Rarely | Never |
| --- | --- | --- | --- | --- | --- | --- |

20. How often in the last two weeks have you felt bloating in your belly?

| Almost | Almost always | Often | Few times | Very few times | Rarely | Never |
| --- | --- | --- | --- | --- | --- | --- |

21. How often in the last two weeks have you felt calm and relaxed?

| Almost | Almost always | Often | Few times | Very few times | Rarely | Never |
| --- | --- | --- | --- | --- | --- | --- |

22. How often in the last two weeks have you had problems with rectal bleeding with your bowel movements?

| Almost | Almost always | Often | Few times | Very few times | Rarely | Never |
| --- | --- | --- | --- | --- | --- | --- |

23. How much of the time in the last two weeks have you felt ashamed because of your intestinal problem?

| Almost | Almost always | Often | Few times | Very few times | Rarely | Never |
| --- | --- | --- | --- | --- | --- | --- |

24. How much of the time, in the last two weeks, were you bothered by having to go to the bathroom to have a bowel movement and were unable to do so, despite your effort?

| Almost | Almost always | Often | Few times | Very few times | Rarely | Never |
| --- | --- | --- | --- | --- | --- | --- |

25. How much of the time in the last two weeks have you felt like crying?

| Almost | Almost always | Often | Few times | Very few times | Rarely | Never |
| --- | --- | --- | --- | --- | --- | --- |

26. How much of the time in the last two weeks have you been bothered by accidentally having a bowel movement in your pants?

| Almost | Almost always | Often | Few times | Very few times | Rarely | Never |
| --- | --- | --- | --- | --- | --- | --- |

27. How much of the time in the last two weeks have you felt angry because of your intestinal problem?

| Almost | Almost always | Often | Few times | Very few times | Rarely | Never |
| --- | --- | --- | --- | --- | --- | --- |

28. How much has your sexual activity decreased in the last two weeks because of your intestinal problem?

| Absolutely no sex | Big limitation | Moderate limitation | Some limitation | Little limitation | Rarely limitation | No limitations whatsoever |
| --- | --- | --- | --- | --- | --- | --- |

29. How long in the last two weeks have you felt sick?

| Almost | Almost always | Often | Few times | Very few times | Rarely | Never |
| --- | --- | --- | --- | --- | --- | --- |

30. How long in the last two weeks have you felt irritable?

| Almost | Almost always | Often | Few times | Very few times | Rarely | Never |
| --- | --- | --- | --- | --- | --- | --- |

31. How long in the last two weeks have you felt a lack of understanding from other people?

| Almost | Almost always | Often | Few times | Very few times | Rarely | Never |
| --- | --- | --- | --- | --- | --- | --- |

32. How satisfied, happy or grateful have you felt with your personal life in the last two weeks?

| Very dissatisfied | Generally dissatisfied | A little dissatisfied | Generally satisfied | Satisfied | Very satisfied | Extremely satisfied |
| --- | --- | --- | --- | --- | --- | --- |
