# Supplementary material for: Association between Ulcerative Colitis, mental health, and Quality of Life in a Pakistani cohort: A cross-sectional analysis
Source: PLoS One. 2026 Aug 21;21(8):e0356531. doi: 10.1371/journal.pone.0356531 (PMC13496436; doi:10.1371/journal.pone.0356531)
Supplement: Supplementary file 2 [file pone.0356531.s002.docx]

Inclusivity in global research

PLOS’ policy on inclusivity in global research aims to improve transparency in the reporting of research performed outside of researchers’ own country or community and ensures that PLOS publications reporting global research adhere to high standards for research ethics and authorship. Authors of relevant research articles may be asked to complete the questionnaire below, which outlines ethical, cultural, and scientific considerations specific to inclusivity in global research. This questionnaire may be requested when researchers have travelled to a different country to conduct research, if research uses samples collected in another country, research with Indigenous populations or their lands, or if research is on cultural artefacts. Researchers travelling to another country solely to use laboratory equipment will not normally be required to complete the questionnaire. However, the questionnaire can be requested at the journal’s discretion for any submission – if you have been requested to complete this questionnaire by the PLOS journal you submitted to, please do so.

Please complete the questionnaire below and include this as a Supporting Information file with your manuscript. Note that if your paper is accepted for publication, this checklist will be published with your article in the supporting information files. Please ensure that you reference the checklist in the main body of your manuscript. We suggest adding a subsection ‘Inclusivity in global research’ to your Methods section and adding the following sentence: “Additional information regarding the ethical, cultural, and scientific considerations specific to inclusivity in global research is included in the Supporting Information (SX Checklist)”

The questions have been designed to be applicable to a wide range of study types, and there are subsections for both human subjects research and non-human subjects research. If any of the questions are not relevant to your research please mark them as “N/A” as appropriate.

**Ethical considerations, permits and authorship**

*This section is applicable to all research types.*

Provide details as to who granted permissions and/or consent for the study to take place in the Methods section of your manuscript. This should include the names of **all** ethics boards, governmental organizations, community leaders or other bodies that provided approval for the study. If individuals provided approval refer to these people by their role or title but do not list their name(s).

*Reported on page number: 5 (Methods: Study design and setting / Study population and subjects)*

**Response:** The study was approved by the Institutional Research and Ethics Forum of Holy Family Hospital, Rawalpindi (protocol approval number 851/IREF/RMU/2024), and by the Institutional Ethical Review Board and Bio-Ethical Committee (BEC) of Quaid-i-Azam University, Islamabad (protocol approval number BEC-FBS-QAU2023-574). Written informed consent was obtained from each participant before inclusion. The study was conducted in accordance with the Declaration of Helsinki, and all participant data were entered and stored anonymously without personal identifiers.

If there were any deviations from the study protocol after approval was obtained please provide details of these changes in the Methods section of your manuscript.
Did this study involve local collaborators that are residents of the country where the research was conducted or members of the community studied? If you do not have any authors from said communities, please provide an explanation for this below.
Everyone listed as an author should meet PLOS’ criteria for authorship and all individuals who meet these criteria should be included in the author byline, rather than the acknowledgements. For further information please see the journal’s Authorship Policy.

*Reported on the Title page / Declarations*

**Response:** All listed authors meet the authorship criteria stated in the manuscript. The authors' confirmation states that the manuscript has been read and approved by all authors. The contribution statement specifies that all authors contributed substantially to conception and design; SS, AK, and TSA collected data; SM, AK, SA, and BA contributed to data acquisition and analysis; SM and SS drafted the manuscript; AK, TSA, SA, BA, YD, WF, and YF critically revised the manuscript for important intellectual content; and AK and YF supervised the study. This supports transparent inclusion of both local Pakistani contributors and international collaborators in the byline rather than only in acknowledgements.

*Reported on page numbers: 1, 2, 5-7*

**Response:** Yes. The study involved local collaborators from Pakistan, including authors affiliated with Quaid-i-Azam University, Islamabad; Holy Family Hospital, Rawalpindi; and Islamabad Medical & Dental College. Local collaborators played an essential role in facilitating participant recruitment, coordinating with the clinical setting, and supporting data collection from patients with ulcerative colitis in Pakistan. The local clinical and academic institutions involved in the study, including Holy Family Hospital, Rawalpindi, and Quaid-i-Azam University, Islamabad, are clearly mentioned in the manuscript, along with the relevant ethics approvals. The study design and overall conceptual framework were developed with substantial input from the corresponding authors and the first/co-first authors. The corresponding authors provided academic supervision, methodological guidance, and critical intellectual input throughout the study. The first/co-first authors contributed substantially to the conception of the work, data organization, analysis, manuscript drafting, and interpretation of findings. In particular, Dr. Saima Mushtaq contributed to manuscript write-up, data analysis, interpretation of results, and revision of the manuscript. Other local collaborators contributed to data collection, clinical coordination, data acquisition, and critical revision of the manuscript.All authors listed in the manuscript made substantial contributions according to the journal’s authorship policy, including contributions to study conception/design, data collection, analysis and interpretation, drafting or critical revision of the manuscript, and approval of the final version. The author list therefore appropriately reflects the scientific, clinical, and local contributions made to the study.

*Reported on page number: N/A - no deviations reported*

**Response:** No deviations from the approved study protocol were reported after ethics approval was obtained. The study procedures, participant eligibility criteria, consent process, anonymized data handling, and questionnaire-based assessments were conducted as described in the approved protocol and Methods section.

**Human subjects research (e.g. health research, medical research, cross-cultural psychology)**

Did you obtain written informed consent from a representative of the local community or region before the research took place? How did you establish who speaks for the community? Details of written informed consent obtained from study participants should be reported separately in the Methods section of your manuscript.

*Reported on page numbers: 5-7 (Methods and Ethics statement)*

**Response:** This was hospital-based human subjects health research involving adult ulcerative colitis patients attending outpatient IBD clinics, rather than research requiring permission from a traditional community leader. Permission to conduct the research was obtained from the relevant local institutional authorities: the Institutional Research and Ethics Forum of Holy Family Hospital, Rawalpindi, where recruitment took place, and the Institutional Ethical Review Board and Bio-Ethical Committee of Quaid-i-Azam University, Islamabad. These bodies were the appropriate local institutional authorities for approving clinical research in this setting. Written informed consent was obtained separately from each participant before enrolment.

How did members of the local community provide input on the aims of the research investigation, its methodology, and its anticipated outcome(s)?

*Reported on page number: 1, 5-7 (Introduction and Methods)*

**Response:** The study was shaped by local Pakistani clinical and academic collaborators who work directly in the setting where the research was conducted and who understand the clinical, cultural, and service-delivery challenges faced by Pakistani patients with ulcerative colitis. The local team contributed to the study aims, which addressed a locally under-recognized gap: anxiety, depression, and quality of life among UC patients in Pakistan. Local collaborators also contributed to the methodology, including clinic-based recruitment, face-to-face data collection, use of validated psychological and quality-of-life instruments, Urdu explanation of questionnaire items when needed, and interpretation of the findings in relation to stigma, limited mental health awareness, and barriers to integrated care in Pakistan. The anticipated outcome was to inform culturally sensitive screening and holistic UC management in the local clinical context.

When engaging with the local community, how did you ensure that the informed consent documents and other materials could be understood by local stakeholders?

*Reported on page number: 6-7 (Methods: Data collection method / Limitations)*

**Response:** The study used face-to-face interviews conducted during patient visits by the local research team. Written informed consent was obtained prior to inclusion. To minimize comprehension barriers, questionnaires and study materials were explained in Urdu when necessary by the primary investigator/local research team. Validated Urdu versions of HADS and BDI-II were used, and the IBDQ-32 was administered with permission. No personal identifiers were collected in the analytical dataset, which was explained to participants as part of the consent and confidentiality process.

Will the findings of the research be made available in an understandable format to stakeholders in the community where the study was conducted (e.g. via a presentation, summary report, copies of publications, etc.)? Please provide details of how this will be achieved.

*Reported on page number: 7 and 14 (Planned dissemination)*

**Response:** Yes. The findings will be shared with stakeholders in understandable formats after publication or final journal decision. The corresponding/local investigators will provide copies of the publication or a plain-language summary to the collaborating clinical department and pharmacy/IBD clinic staff at Holy Family Hospital, Rawalpindi, and to relevant academic collaborators at Quaid-i-Azam University. The results may also be presented in departmental meetings, student/research seminars, and clinical discussions focused on integrated care for UC patients. Where appropriate, a short patient-friendly summary can be made available through the local clinical team to explain the importance of mental health screening, quality-of-life assessment, treatment adherence, and timely referral for psychological support.

**Non-human subjects research using specimens/ animals collected as part of the study, or those housed in archival collections. Examples include archaeology, paleontology, botany and zoology.**

Did the permission you obtained from a local authority to perform the study include an agreement on access to outputs and benefit sharing? This may include procedures to enable fair distribution of the benefits and resources arising from the research performed. Please include any details of Prior Informed Consent and Benefit Sharing Agreements obtained. These may be required by field-specific regulations, for example the Convention on Biological Diversity (CBD) and the associated Nagoya Protocol.

*Reported on page number: N/A*

**Response:** N/A. This was human subjects clinical research involving questionnaire-based and clinical data collection from ulcerative colitis patients. It did not involve non-human specimens, animals, archaeology, paleontology, botany, zoology, Indigenous lands, cultural artefacts, genetic resources, or material governed by the Convention on Biological Diversity/Nagoya Protocol.

If the material used in your study was imported, please A) provide the year it was imported and B) indicate whether permits were obtained to import/export the materials used, C) provide details of any permits obtained. If this information is not available, please indicate this.

*Reported on page number: N/A*

**Response:** N/A. No biological material, animal material, archaeological material, cultural artefacts, or archival specimens were imported or exported for this study. The study used anonymized clinical and questionnaire data collected in Pakistan.

If you used archival specimens, please state how the material used in your study was acquired by the institute it is held in and provide details of any permits obtained for the original excavations/ sample collection. If this information is not available, please indicate this.

*Reported on page number: N/A*

**Response:** N/A. No archival specimens were used.

How was the potential cultural significance of the materials collected in your study to local communities considered in your research design? Were Indigenous peoples and/or local researchers and institutions involved with archaeological excavations / collection of specimens? If so, please provide a description of their involvement.

*Reported on page number: 5-7 (N/A for specimens; addressed under Human subjects research)*

**Response:** N/A for materials/specimens, as no cultural artefacts, Indigenous lands, archaeological excavations, or non-human specimens were involved. Cultural considerations were nevertheless relevant to the human-subjects component of the study. The research design considered stigma around mental health, gastrointestinal symptoms, and health-seeking behavior in Pakistan by involving local Pakistani clinical and academic collaborators, using face-to-face interviews, explaining questionnaire items in Urdu where necessary, maintaining participant anonymity, and interpreting the findings within the local clinical and cultural context.

If your manuscript includes photographs of human remains please indicate whether authors obtained permission from descendants or affiliated cultural communities to do so.

*Reported on page number: N/A*

**Response:** N/A. The manuscript does not include photographs of human remains.
