# Supplementary material for: Association between Ulcerative Colitis, mental health, and Quality of Life in a Pakistani cohort: A cross-sectional analysis
Source: PLoS One. 2026 Aug 21;21(8):e0356531. doi: 10.1371/journal.pone.0356531 (PMC13496436; doi:10.1371/journal.pone.0356531)
Supplement: Supplementary file 3 [file pone.0356531.s004.docx]

**Author’s response to reviewers’ comments** (Manuscript title: Impact of Ulcerative Colitis on Mental Health and Life Quality in a Pakistani Cohort)

**Dear Editor,**

Thank you for allowing us to submit a revised draft of our letter. I have attached a revised version of our manuscript, Impact of Ulcerative Colitis on Mental Health and Life Quality in a Pakistani Cohort, for evaluation and publication in PLOS One. We appreciate the time and effort you and the reviewers have dedicated to providing valuable feedback on our manuscript.

**General Response**

We are thankful to the reviewers for evaluating our manuscript. All the suggestions have been incorporated into the revised manuscript. The reviewers’ comments were constructive and included updated information. We hope these changes will clarify points and improve the overall presentation of our manuscript.

Here is a point-by-point response to the reviewers’ comments and concerns.

**Reviewer 1**

This manuscript addresses an important and clinically relevant topic: the psychological burden and quality of life among patients with ulcerative colitis in a low- and middle-income setting. The integration of disease severity (Mayo score), disease extent (Montreal classification), and validated psychological instruments (HADS, BDI-II, IBDQ-32) is commendable and adds multidimensional value to the study. However, several methodological and reporting issues need clarification before the manuscript can be considered technically sound.
Response: We are grateful for the careful evaluation of our manuscript and for the constructive feedback. All suggested revisions have been addressed in the revised version as outlined below:

First, there are inconsistencies in the reported data that require careful reconciliation. The distribution of disease extent (E1, E2, E3) reported in the Results section differs from that presented in Table 2. These values should be cross-checked and corrected to ensure internal consistency throughout the manuscript.
Response: We thank the reviewer for carefully identifying this inconsistency. Upon re-evaluation, we found that the discrepancy occurred due to an inadvertent typographical/reporting error in the text. The values presented in the table are correct and have now been consistently updated throughout the manuscript to ensure internal accuracy and uniformity between the Results section and Table 2.

Second, the graphical presentation of the Mayo score components (Figure 1) does not appear to sum to the total sample size (n = 101). For example, the counts shown for stool frequency and other components are lower than expected. It is unclear whether this reflects missing data, a subgroup analysis, or a graphical error. The authors should clarify whether any observations were excluded from these analyses and explicitly report missing values if present.
Response: We thank the reviewer for this important observation. The values presented in Figure 1 represent the breakdown of individual Mayo score component categories (e.g., stool frequency, rectal bleeding, endoscopic findings, and physician global assessment) rather than the overall presenting symptoms of all 101 UC patients. Therefore, the frequencies shown in each component reflect the distribution across severity categories within that specific Mayo score parameter and are not intended to sum directly to the total sample size. The methodology for Mayo score calculation has already been clearly described in the manuscript (Line 146 and Table 1). No observations were excluded from this analysis, and there were no additional missing values specifically related to the Mayo score components.

Third, in Figure 2 (correlation heatmap), p-values are displayed for self-correlations (ρ = 1.00). Since correlation of a variable with itself is structurally 1.00 and does not require hypothesis testing, reporting p-values in these diagonal cells is not statistically appropriate. Additionally, p-values reported in the heatmap should be checked for consistency with those presented in Table 4.
Response: We thank the reviewer for this important statistical observation. We agree that self-correlations (ρ = 1.00) are structural values and do not require hypothesis testing; therefore, the p-values displayed in the diagonal cells of Figure 2 were unnecessary. The figure has now been revised to remove p-values from self-correlation cells. In addition, all p-values presented in the heatmap have been carefully cross-checked and updated for consistency with the corresponding values reported in Table 4.

Fourth, the reported mean scores for HADS-A, HADS-D, and BDI-II appear unusually low relative to the proportion of participants categorized as borderline or abnormal. The authors should verify that the mean values correspond to the full scale ranges (HADS 0–21 per subscale; BDI-II 0–63) and confirm that no transformation or rescaling was applied. If any scoring modification was used, this must be clearly described in the Methods section.
Response: We thank the reviewer for carefully identifying this point. The reported mean values represent categorized scoring distributions used for analytical grouping rather than raw cumulative questionnaire scores across the full scale ranges. No transformation or rescaling of HADS-A, HADS-D, or BDI-II scores was applied. The proportions of borderline and abnormal categories were calculated according to the standard, validated cut-off criteria for each instrument; therefore, they were reported as 1.68 or 1.49. I hope this may have cleared the confusion.

Fifth, regarding the multivariable regression analysis, several closely related psychological measures (HADS-D and BDI-II) were included simultaneously. The authors should clarify whether multicollinearity diagnostics (e.g., VIF) were assessed and report model assumption checks (normality of residuals, homoscedasticity) to support the validity of the linear model.
Response: We thank the reviewer for this valuable methodological comment. Prior to conducting the multivariable regression analysis, multicollinearity diagnostics were assessed to ensure the suitability of including related psychological variables such as HADS-D and BDI-II within the same model and from that all variables were found to be within acceptable limits, indicating the absence of significant multicollinearity. In addition, standard linear regression assumptions, including normality of residuals, linearity, independence, and homoscedasticity, were evaluated through residual plots and diagnostic testing, with no major violations observed. As the regression analysis was primarily exploratory and due to manuscript word limit constraints, detailed diagnostic outputs were not presented in the main text; however, the model assumptions were adequately satisfied to support the validity and interpretation of the reported findings and is now added in line 186 for clear understanding.

In addition, the Data Availability statement should be harmonized. The manuscript text indicates that further inquiries can be directed to the corresponding author, whereas the submission form states that all relevant data are included in the manuscript and Supporting Information files. These statements should be aligned to comply fully with journal data policy.
Response: We thank the reviewer for highlighting this inconsistency. The Data Availability statement has now been updated and harmonized in both sections to ensure consistency and compliance with the journal’s data-sharing policy.

Finally, while the discussion appropriately acknowledges limitations such as the cross-sectional design and single-center sampling, some interpretations should remain strictly associative rather than suggest directional implications.
Response: We thank the reviewer for this valuable observation. The limitations related to the cross-sectional design and single-center sampling have been acknowledged in the manuscript, followed by future prospects, emphasizing that the study findings can be further strengthened and expanded if followed in future.

Overall, the study addresses an important gap and has the potential to contribute meaningful data from an underrepresented population. However, the issues outlined above—particularly those related to data consistency and statistical reporting—should be carefully addressed to ensure methodological transparency and reproducibility.

**Reviewer 2**

The use of convenience sampling in this study represents a major methodological limitation that directly impacts the validity of the inferential statistical analyses performed. While the authors employed ANOVA, t-tests, and regression analyses, these methods assume that the sample is drawn from a probability-based population, which is not the case here. As a result, the reported p-values and statistical significance cannot be interpreted as evidence of population-level effects.While the topic is important and the dataset has potential value, the current analytical framework is fundamentally incompatible with the sampling design, rendering the conclusions unreliable. Therefore, I recommend rejection in its current form.

Response: We respectfully thank the reviewer for this important methodological concern. We acknowledge that convenience sampling may limit external validity and population-level generalizability, and this limitation has already been explicitly recognized in the manuscript. However, the primary objective of the present study was exploratory and hypothesis-generating in nature, aimed at assessing associations between disease severity, psychological distress, and QoL among UC patients attending a tertiary care center in Pakistan, where such data remain limited.

The inferential statistical methods employed, including correlation analysis, group comparisons, and regression modeling, were used to evaluate internal associations within the studied cohort rather than to claim definitive population-level causation or national prevalence estimates. Similar analytical approaches have been widely used in observational hospital-based studies utilizing non-probability sampling, particularly in clinical and psychosocial research settings.

We have clarified that findings should be interpreted within the context of the sampled population and should not be generalized without confirmation from larger multicenter probability-based studies. Future prospective and multicenter investigations have also been recommended to validate and extend these findings. We appreciate your keen interest in reviewing the manuscript and hope that the mentioned clarification will be helpful in understanding the main theme.

On behalf of all authors, I would like to thank the reviewers for their valuable time reviewing this manuscript. If there are still any queries, we will happily respond and cooperate.

Kind Regards,

Research Team

Amjad Khan, PhD (Corresponding Author)

E-mail: amjadkhan@qau.edu.pk
