# Supplementary material for: Association between Ulcerative Colitis, mental health, and Quality of Life in a Pakistani cohort: A cross-sectional analysis
Source: PLoS One. 2026 Aug 21;21(8):e0356531. doi: 10.1371/journal.pone.0356531 (PMC13496436; doi:10.1371/journal.pone.0356531)
Supplement: Supplementary file 4 [file pone.0356531.s005.docx]

**Author’s response to reviewers’ comments** (Manuscript title: Association Between Ulcerative Colitis, Mental Health, and Quality of Life in a Pakistani Cohort: A Cross-Sectional Analysis)

**Dear Editor,**

Thank you for allowing us to submit a revised draft of our letter. I have attached a revised version of our manuscript, Association Between Ulcerative Colitis, Mental Health, and Quality of Life in a Pakistani Cohort: A Cross-Sectional Analysis, for evaluation and publication in PLOS One. We appreciate the time and effort you and the reviewers have dedicated to providing valuable feedback on our manuscript.

**General Response**

We are thankful to the reviewers for evaluating our manuscript. All the suggestions have been incorporated into the revised manuscript. The reviewers’ comments were constructive and included updated information. We hope these changes will clarify points and improve the overall presentation of our manuscript.

Here is a point-by-point response to the reviewers’ comments and concerns.

**Reviewer 1**

The reported mean values for HADS-A, HADS-D, and BDI-II appear to represent categorized scores rather than the original questionnaire scores. To avoid potential confusion for readers, please clarify this explicitly in the Methods and Results sections and indicate whether these values reflect category coding or raw scale scores.
With this minor clarification, I believe the manuscript is suitable for publication.
Response: We sincerely thank the reviewer for this constructive comment and for the positive evaluation of our manuscript. As suggested, we have revised the Methodology section (page 7, line 188) to clarify that the reported mean values for HADS-A, HADS-D, and BDI-II represent category-coded scores used in statistical analyses rather than the original cumulative raw questionnaire scores. We have also stated that no transformation or rescaling of the original questionnaire scores was performed, and that participant classification into normal, borderline, abnormal, or severity categories was based on the standard validated cut-off values for each instrument. We appreciate the reviewer's valuable suggestion, which has improved the manuscript's clarity and interpretability.

**Reviewer 2**

The term “impact” may imply a causal relationship between ulcerative colitis and the assessed outcomes. However, given the cross-sectional design of the study, causality cannot be established, as exposure and outcomes are assessed simultaneously. Therefore, from a methodological perspective, a title referring to an "association" or "relationship" rather than an "impact" would be more appropriate. For example: Mental Health and Quality of Life Among Patients with Ulcerative Colitis: A Cross-Sectional Study in a Pakistani Cohort or Association Between Ulcerative Colitis, Mental Health, and Quality of Life in a Pakistani Cohort or Assessment of Mental Health and Quality of Life in Patients with Ulcerative Colitis: A Cross-Sectional Analysis

Response: We sincerely thank the reviewer for this valuable suggestion. The manuscript title has been revised and updated in accordance with your recommendation to more accurately reflect the scope, objectives, and key findings of the study. We appreciate your constructive feedback, which has improved the title's clarity and relevance.

Line:117-118 The sentence "In Pakistan, the prevalence of UC appears to be increasing (9), likely due to lifestyle changes, urbanization, dietary shifts, and better diagnostic capacities. " is somewhat misleading, as diagnostic capacity affects disease detection and the estimation of prevalence rather than necessarily contributing to a true increase in disease occurrence. In this case, it would be more appropriate to replace the sentence with the following: "In Pakistan, the prevalence of UC appears to be increasing (9), potentially as a result of lifestyle changes, urbanization, and dietary transitions. In addition, advances in diagnostic capabilities may have improved case detection, thereby enhancing the accuracy of prevalence estimates"

Response: We thank the reviewer for the careful evaluation of our manuscript. The suggested revision has been addressed, and the manuscript has been comprehensively updated.

Methodology:
1. I understand that the study population consisted of Punjabi participants, as the authors state that the questionnaires were translated into Urdu. This information should be clearly specified in the Materials section.

Response: We thank the reviewer for this observation. We would like to clarify that the study population consisted of Pakistani patients, not exclusively Punjabi participants. Urdu is the national language of Pakistan and is widely understood across all provinces, irrespective of participants' native languages. Therefore, the validated Urdu versions of the questionnaires were used to ensure standardized administration and facilitate comprehension among all participants.

2. Consider simplifying the description accompanying Table 1. to improve clarity and readability.

Response: We thank the reviewer for this recommendation. The description accompanying Table 1 presents only the individual components of the Mayo score used to assess disease activity, rather than the baseline clinical characteristics or presenting symptoms of the study participants (page 6, lines 146-153).

3. The authors should also specify the version of the Declaration of Helsinki referenced (e.g., the Declaration of Helsinki, 1975, revised in 2000).

Response: We thank the reviewer for this helpful suggestion. The Ethics Statement has been updated to specify the version of the Declaration of Helsinki followed in this study (page 5, line 142).

4. Additionally, demographic information on the participants’ age is lacking and should be included.

Response: We thank the reviewer for this comment. We would like to clarify that the demographic information on participants' ages has already been reported in the first paragraph of the Results section of the manuscript (Page 8, Line 207).

5. The manuscript does not clearly indicate whether the patients with UC had any additional chronic diseases or comorbid conditions.

Response: We thank the reviewer for this important observation. We would like to clarify that patients with additional chronic diseases or significant comorbidities were excluded per the study eligibility criteria to minimize potential confounding. This has now been explicitly stated in the methodology to improve clarity regarding the study population.

6. The manuscript lacks information on the therapies received by the patients enrolled in the study, which may be relevant for the interpretation of the results.

Response: We thank the reviewer for this important comment. We acknowledge that treatment may influence psychological well-being and QoL in patients with UC. However, the primary objective of the present study was to evaluate the association between disease severity and psychological outcomes, rather than treatment effectiveness. Since patients were receiving heterogeneous treatment regimens based on their individual clinical status, disease severity, and the treating physician's judgment, meaningful comparison of therapeutic effects was beyond the scope of this study and could have introduced substantial heterogeneity. Therefore, treatment-related analyses were not performed. We have clarified this point and acknowledged it as a limitation, and we recommend that future prospective studies investigate the impact of specific therapeutic regimens on psychological distress and QoL in UC patients.

7. The methodology section requires linguistic revision and simplification to enhance readability and comprehension.

Response: We are grateful to the reviewer for the insightful comments and recommendations. The manuscript has been revised accordingly, and all relevant sections have been updated to address the concerns raised. We appreciate the valuable feedback, which has strengthened the overall quality of the manuscript.

Discussion.
1. The first paragraph of the Discussion would benefit from revision and condensation. In addition, the core of the Discussion should provide a more in-depth discussion of the results in the context of the existing literature, particularly studies conducted by other authors addressing the same research topic, eg. doi: 10.1159/000538618; doi: 10.1159/000528788; doi: 10.1097/PG9.0000000000000287)

Response: We sincerely thank the reviewer for this constructive suggestion. In response, we have substantially revised the Discussion section by condensing the introductory paragraph to improve focus and readability.

Major issue:
2. A major limitation that undermines the reliability of the conclusions is the small sample size, particularly given that all participants were recruited from a single center. This substantially limits the generalizability of the findings and does not allow for drawing conclusions applicable to the entire Pakistani population. The authors should acknowledge this limitation more explicitly and interpret their results with greater caution.

Response: We thank the reviewer for this important observation. The limitation regarding the relatively small sample size has now been explicitly acknowledged in the limitations section (page 12, line 351). We have clarified that the modest sample size, together with the single-center design, may limit the generalizability of the findings. This has also been highlighted as an area for future research, with recommendations for larger, multicenter studies to validate and extend the present findings.

3. In addition, I suggest revising and shortening the first paragraph of the Discussion section. The main body of the Discussion should be expanded to provide a more comprehensive comparison of the findings with those reported by other authors investigating this topic.

Response: We sincerely thank the reviewer for this valuable suggestion. The Discussion section has been comprehensively revised and updated to improve its structure, reduce repetition, and provide a more focused interpretation of the findings. Additional comparisons with relevant published literature have been incorporated to better contextualize the results, thereby strengthening the scientific discussion and the manuscript's overall quality.

Minor issue:
1) It is generally recommended to avoid starting sentences with abbreviations.

Response: We thank the reviewer for this helpful suggestion. The manuscript has been revised accordingly, and sentences beginning with abbreviations have been rephrased to improve readability and conform to standard scientific writing conventions.

2) There are several inconsistencies in the formatting of the reference list that should be corrected to ensure compliance with the journal's guidelines.

Response: We thank the reviewer for this valuable observation. The reference list has been carefully reviewed and updated using reference management software to ensure consistent formatting, accurate citations, and compliance with the journal's referencing style. All in-text citations and corresponding references have been cross-checked and harmonized throughout the manuscript.

3) A thorough language revision is recommended to improve clarity, readability, and grammatical accuracy.

Response: We thank the reviewer for the careful evaluation of our manuscript. The suggested revisions have been addressed, and the manuscript has been comprehensively updated.

4) The term “race” should preferably be replaced with “ethnic origin” (or “ethnicity”), as it is more appropriate and precise in this context.

Response: We are grateful to the reviewer for the insightful recommendations, and we have updated all relevant sections to address the concerns raised.

On behalf of all authors, I would like to thank the reviewers for their valuable time reviewing this manuscript. If there are still any queries, we will happily respond and cooperate.

Kind Regards,

Research Team

Amjad Khan, PhD (Corresponding Author)

E-mail: amjadkhan@qau.edu.pk
