# Supplementary material for: Association between Ulcerative Colitis, mental health, and Quality of Life in a Pakistani cohort: A cross-sectional analysis
Source: PLoS One. 2026 Aug 21;21(8):e0356531. doi: 10.1371/journal.pone.0356531 (PMC13496436; doi:10.1371/journal.pone.0356531)
Supplement: Peer review history

# Peer review: Decision Letter 0

**DOI:** 10.1371/journal.pone.0356531.r001

**Authors:** Lubna Shirin

2 Apr 2026

Dear Dr.  Khan,

Thank you for submitting your manuscript to PLOS ONE. After careful consideration, we feel that it has merit but does not fully meet PLOS ONE’s publication criteria as it currently stands. Therefore, we invite you to submit a revised version of the manuscript that addresses the points raised during the review process.

Please submit your revised manuscript by May 17 2026 11:59PM. If you will need more time than this to complete your revisions, please reply to this message or contact the journal office at plosone@plos.org. When you're ready to submit your revision, log on to https://www.editorialmanager.com/pone/ and select the 'Submissions Needing Revision' folder to locate your manuscript file.

- A letter that responds to each point raised by the academic editor and reviewer(s). You should upload this letter as a separate file labeled 'Response to Reviewers'.
- A marked-up copy of your manuscript that highlights changes made to the original version. You should upload this as a separate file labeled 'Revised Manuscript with Track Changes'.
- An unmarked version of your revised paper without tracked changes. You should upload this as a separate file labeled 'Manuscript'.

If you would like to make changes to your financial disclosure, please include your updated statement in your cover letter. Guidelines for resubmitting your figure files are available below the reviewer comments at the end of this letter.

If applicable, we recommend that you deposit your laboratory protocols in protocols.io to enhance the reproducibility of your results. Protocols.io assigns your protocol its own identifier (DOI) so that it can be cited independently in the future. For instructions see: https://journals.plos.org/plosone/s/submission-guidelines#loc-laboratory-protocols. Additionally, PLOS ONE offers an option for publishing peer-reviewed Lab Protocol articles, which describe protocols hosted on protocols.io. Read more information on sharing protocols at https://plos.org/protocols?utm_medium=editorial-email&utm_source=authorletters&utm_campaign=protocols.

As the corresponding author, your ORCID iD is verified in the submission system and will appear in the published article. PLOS supports the use of ORCID, and we encourage all coauthors to register for an ORCID iD and use it as well. Please encourage your coauthors to verify their ORCID iD within the submission system before final acceptance, as unverified ORCID iDs will not appear in the published article. *Only*  the individual author can complete the verification step; PLOS staff *cannot*  verify ORCID iDs on behalf of authors.

We look forward to receiving your revised manuscript.

Kind regards,

Lubna Shirin, Ph.D, M.Phil, MBBS

Academic Editor

PLOS One

Journal Requirements:

When submitting your revision, we need you to address these additional requirements.

1.Please ensure that your manuscript meets PLOS ONE's style requirements, including those for file naming. The PLOS ONE style templates can be found at

https://journals.plos.org/plosone/s/file?id=wjVg/PLOSOne_formatting_sample_main_body.pdf and

https://journals.plos.org/plosone/s/file?id=ba62/PLOSOne_formatting_sample_title_authors_affiliations.pdf

2. Please include a complete copy of PLOS’ questionnaire on inclusivity in global research in your revised manuscript. Our policy for research in this area aims to improve transparency in the reporting of research performed outside of researchers’ own country or community. The policy applies to researchers who have travelled to a different country to conduct research, research with Indigenous populations or their lands, and research on cultural artefacts. The questionnaire can also be requested at the journal’s discretion for any other submissions, even if these conditions are not met.  Please find more information on the policy and a link to download a blank copy of the questionnaire here: https://journals.plos.org/plosone/s/best-practices-in-research-reporting. Please upload a completed version of your questionnaire as Supporting Information when you resubmit your manuscript.

3. We note that your Data Availability Statement is currently as follows: “All relevant data are within the manuscript and its Supporting Information files.”

Please confirm at this time whether or not your submission contains all raw data required to replicate the results of your study. Authors must share the “minimal data set” for their submission. PLOS defines the minimal data set to consist of the data required to replicate all study findings reported in the article, as well as related metadata and methods (https://journals.plos.org/plosone/s/data-availability#loc-minimal-data-set-definition).

For example, authors should submit the following data:

- The values behind the means, standard deviations and other measures reported;

- The values used to build graphs;

- The points extracted from images for analysis.

Authors do not need to submit their entire data set if only a portion of the data was used in the reported study.

If your submission does not contain these data, please either upload them as Supporting Information files or deposit them to a stable, public repository and provide us with the relevant URLs, DOIs, or accession numbers. For a list of recommended repositories, please see https://journals.plos.org/plosone/s/recommended-repositories.

If there are ethical or legal restrictions on sharing a de-identified data set, please explain them in detail (e.g., data contain potentially sensitive information, data are owned by a third-party organization, etc.) and who has imposed them (e.g., an ethics committee). Please also provide contact information for a data access committee, ethics committee, or other institutional body to which data requests may be sent. If data are owned by a third party, please indicate how others may request data access.

4. We notice that your supplementary figures are uploaded with the file type 'Figure'. Please amend the file type to 'Supporting Information'. Please ensure that each Supporting Information file has a legend listed in the manuscript after the references list.

5. Your ethics statement should only appear in the Methods section of your manuscript. If your ethics statement is written in any section besides the Methods, please move it to the Methods section and delete it from any other section. Please ensure that your ethics statement is included in your manuscript, as the ethics statement entered into the online submission form will not be published alongside your manuscript.

6. If the reviewer comments include a recommendation to cite specific previously published works, please review and evaluate these publications to determine whether they are relevant and should be cited. There is no requirement to cite these works unless the editor has indicated otherwise.

[Note: HTML markup is below. Please do not edit.]

Reviewer's Responses to Questions

**Comments to the Author**

1. Is the manuscript technically sound, and do the data support the conclusions?

Reviewer #1: No

Reviewer #2: No

**********

2. Has the statistical analysis been performed appropriately and rigorously?

Reviewer #1: No

Reviewer #2: No

**********

3. Have the authors made all data underlying the findings in their manuscript fully available?

The PLOS Data policy

Reviewer #1: No

Reviewer #2: Yes

**********

4. Is the manuscript presented in an intelligible fashion and written in standard English?

Reviewer #1: Yes

Reviewer #2: Yes

**********

Reviewer #1: This manuscript addresses an important and clinically relevant topic: the psychological burden and quality of life among patients with ulcerative colitis in a low- and middle-income setting. The integration of disease severity (Mayo score), disease extent (Montreal classification), and validated psychological instruments (HADS, BDI-II, IBDQ-32) is commendable and adds multidimensional value to the study. However, several methodological and reporting issues need clarification before the manuscript can be considered technically sound.

First, there are inconsistencies in the reported data that require careful reconciliation. The distribution of disease extent (E1, E2, E3) reported in the Results section differs from that presented in Table 2. These values should be cross-checked and corrected to ensure internal consistency throughout the manuscript.

Second, the graphical presentation of the Mayo score components (Figure 1) does not appear to sum to the total sample size (n = 101). For example, the counts shown for stool frequency and other components are lower than expected. It is unclear whether this reflects missing data, a subgroup analysis, or a graphical error. The authors should clarify whether any observations were excluded from these analyses and explicitly report missing values if present.

Third, in Figure 2 (correlation heatmap), p-values are displayed for self-correlations (ρ = 1.00). Since correlation of a variable with itself is structurally 1.00 and does not require hypothesis testing, reporting p-values in these diagonal cells is not statistically appropriate. Additionally, p-values reported in the heatmap should be checked for consistency with those presented in Table 4.

Fourth, the reported mean scores for HADS-A, HADS-D, and BDI-II appear unusually low relative to the proportion of participants categorized as borderline or abnormal. The authors should verify that the mean values correspond to the full scale ranges (HADS 0–21 per subscale; BDI-II 0–63) and confirm that no transformation or rescaling was applied. If any scoring modification was used, this must be clearly described in the Methods section.

Fifth, regarding the multivariable regression analysis, several closely related psychological measures (HADS-D and BDI-II) were included simultaneously. The authors should clarify whether multicollinearity diagnostics (e.g., VIF) were assessed and report model assumption checks (normality of residuals, homoscedasticity) to support the validity of the linear model.

In addition, the Data Availability statement should be harmonized. The manuscript text indicates that further inquiries can be directed to the corresponding author, whereas the submission form states that all relevant data are included in the manuscript and Supporting Information files. These statements should be aligned to comply fully with journal data policy.

Finally, while the discussion appropriately acknowledges limitations such as the cross-sectional design and single-center sampling, some interpretations should remain strictly associative rather than suggest directional implications.

Overall, the study addresses an important gap and has the potential to contribute meaningful data from an underrepresented population. However, the issues outlined above—particularly those related to data consistency and statistical reporting—should be carefully addressed to ensure methodological transparency and reproducibility.

Reviewer #2: The use of convenience sampling in this study represents a major methodological limitation that directly impacts the validity of the inferential statistical analyses performed. While the authors employed ANOVA, t-tests, and regression analyses, these methods assume that the sample is drawn from a probability-based population, which is not the case here. As a result, the reported p-values and statistical significance cannot be interpreted as evidence of population-level effects.While the topic is important and the dataset has potential value, the current analytical framework is fundamentally incompatible with the sampling design, rendering the conclusions unreliable.Therefore, I recommend rejection in its current form.

**********

what does this mean?). If published, this will include your full peer review and any attached files.

If you choose “no”, your identity will remain anonymous but your review may still be made public.

**Do you want your identity to be public for this peer review?**  For information about this choice, including consent withdrawal, please see our Privacy Policy

Reviewer #1: No

Reviewer #2: **Yes:** M Tanveer Hossain Parash

**********

[NOTE: If reviewer comments were submitted as an attachment file, they will be attached to this email and accessible via the submission site. Please log into your account, locate the manuscript record, and check for the action link "View Attachments". If this link does not appear, there are no attachment files.]

To ensure your figures meet our technical requirements, please review our figure guidelines: https://journals.plos.org/plosone/s/figures

You may also use PLOS’s free figure tool, NAAS, to help you prepare publication quality figures: https://journals.plos.org/plosone/s/figures#loc-tools-for-figure-preparation.

NAAS will assess whether your figures meet our technical requirements by comparing each figure against our figure specifications.

# Author comment: Author response to Decision Letter 1

**DOI:** 10.1371/journal.pone.0356531.r002

17 May 2026

Author’s response to reviewers’ comments (Manuscript title: Impact of Ulcerative Colitis on Mental Health and Life Quality in a Pakistani Cohort)

Dear Editor,

Thank you for allowing us to submit a revised draft of our letter. I have attached a revised version of our manuscript, Impact of Ulcerative Colitis on Mental Health and Life Quality in a Pakistani Cohort, for evaluation and publication in PLOS One. We appreciate the time and effort you and the reviewers have dedicated to providing valuable feedback on our manuscript.

General Response

We are thankful to the reviewers for evaluating our manuscript. All the suggestions have been incorporated into the revised manuscript. The reviewers’ comments were constructive and included updated information. We hope these changes will clarify points and improve the overall presentation of our manuscript.

Here is a point-by-point response to the reviewers’ comments and concerns.

Reviewer 1

This manuscript addresses an important and clinically relevant topic: the psychological burden and quality of life among patients with ulcerative colitis in a low- and middle-income setting. The integration of disease severity (Mayo score), disease extent (Montreal classification), and validated psychological instruments (HADS, BDI-II, IBDQ-32) is commendable and adds multidimensional value to the study. However, several methodological and reporting issues need clarification before the manuscript can be considered technically sound.

Response: We are grateful for the careful evaluation of our manuscript and for the constructive feedback. All suggested revisions have been addressed in the revised version as outlined below:

First, there are inconsistencies in the reported data that require careful reconciliation. The distribution of disease extent (E1, E2, E3) reported in the Results section differs from that presented in Table 2. These values should be cross-checked and corrected to ensure internal consistency throughout the manuscript.

Response: We thank the reviewer for carefully identifying this inconsistency. Upon re-evaluation, we found that the discrepancy occurred due to an inadvertent typographical/reporting error in the text. The values presented in the table are correct and have now been consistently updated throughout the manuscript to ensure internal accuracy and uniformity between the Results section and Table 2.

Second, the graphical presentation of the Mayo score components (Figure 1) does not appear to sum to the total sample size (n = 101). For example, the counts shown for stool frequency and other components are lower than expected. It is unclear whether this reflects missing data, a subgroup analysis, or a graphical error. The authors should clarify whether any observations were excluded from these analyses and explicitly report missing values if present.

Response: We thank the reviewer for this important observation. The values presented in Figure 1 represent the breakdown of individual Mayo score component categories (e.g., stool frequency, rectal bleeding, endoscopic findings, and physician global assessment) rather than the overall presenting symptoms of all 101 UC patients. Therefore, the frequencies shown in each component reflect the distribution across severity categories within that specific Mayo score parameter and are not intended to sum directly to the total sample size. The methodology for Mayo score calculation has already been clearly described in the manuscript (Line 146 and Table 1). No observations were excluded from this analysis, and there were no additional missing values specifically related to the Mayo score components.

Third, in Figure 2 (correlation heatmap), p-values are displayed for self-correlations (ρ = 1.00). Since correlation of a variable with itself is structurally 1.00 and does not require hypothesis testing, reporting p-values in these diagonal cells is not statistically appropriate. Additionally, p-values reported in the heatmap should be checked for consistency with those presented in Table 4.

Response: We thank the reviewer for this important statistical observation. We agree that self-correlations (ρ = 1.00) are structural values and do not require hypothesis testing; therefore, the p-values displayed in the diagonal cells of Figure 2 were unnecessary. The figure has now been revised to remove p-values from self-correlation cells. In addition, all p-values presented in the heatmap have been carefully cross-checked and updated for consistency with the corresponding values reported in Table 4.

Fourth, the reported mean scores for HADS-A, HADS-D, and BDI-II appear unusually low relative to the proportion of participants categorized as borderline or abnormal. The authors should verify that the mean values correspond to the full scale ranges (HADS 0–21 per subscale; BDI-II 0–63) and confirm that no transformation or rescaling was applied. If any scoring modification was used, this must be clearly described in the Methods section.

Response: We thank the reviewer for carefully identifying this point. The reported mean values represent categorized scoring distributions used for analytical grouping rather than raw cumulative questionnaire scores across the full scale ranges. No transformation or rescaling of HADS-A, HADS-D, or BDI-II scores was applied. The proportions of borderline and abnormal categories were calculated according to the standard, validated cut-off criteria for each instrument; therefore, they were reported as 1.68 or 1.49. I hope this may have cleared the confusion.

Fifth, regarding the multivariable regression analysis, several closely related psychological measures (HADS-D and BDI-II) were included simultaneously. The authors should clarify whether multicollinearity diagnostics (e.g., VIF) were assessed and report model assumption checks (normality of residuals, homoscedasticity) to support the validity of the linear model.

Response: We thank the reviewer for this valuable methodological comment. Prior to conducting the multivariable regression analysis, multicollinearity diagnostics were assessed to ensure the suitability of including related psychological variables such as HADS-D and BDI-II within the same model and from that all variables were found to be within acceptable limits, indicating the absence of significant multicollinearity. In addition, standard linear regression assumptions, including normality of residuals, linearity, independence, and homoscedasticity, were evaluated through residual plots and diagnostic testing, with no major violations observed. As the regression analysis was primarily exploratory and due to manuscript word limit constraints, detailed diagnostic outputs were not presented in the main text; however, the model assumptions were adequately satisfied to support the validity and interpretation of the reported findings and is now added in line 186 for clear understanding.

In addition, the Data Availability statement should be harmonized. The manuscript text indicates that further inquiries can be directed to the corresponding author, whereas the submission form states that all relevant data are included in the manuscript and Supporting Information files. These statements should be aligned to comply fully with journal data policy.

Response: We thank the reviewer for highlighting this inconsistency. The Data Availability statement has now been updated and harmonized in both sections to ensure consistency and compliance with the journal’s data-sharing policy.

Finally, while the discussion appropriately acknowledges limitations such as the cross-sectional design and single-center sampling, some interpretations should remain strictly associative rather than suggest directional implications.

Response: We thank the reviewer for this valuable observation. The limitations related to the cross-sectional design and single-center sampling have been acknowledged in the manuscript, followed by future prospects, emphasizing that the study findings can be further strengthened and expanded if followed in future.

Overall, the study addresses an important gap and has the potential to contribute meaningful data from an underrepresented population. However, the issues outlined above—particularly those related to data consistency and statistical reporting—should be carefully addressed to ensure methodological transparency and reproducibility.

Reviewer 2

The use of convenience sampling in this study represents a major methodological limitation that directly impacts the validity of the inferential statistical analyses performed. While the authors employed ANOVA, t-tests, and regression analyses, these methods assume that the sample is drawn from a probability-based population, which is not the case here. As a result, the reported p-values and statistical significance cannot be interpreted as evidence of population-level effects.While the topic is important and the dataset has potential value, the current analytical framework is fundamentally incompatible with the sampling design, rendering the conclusions unreliable. Therefore, I recommend rejection in its current form.

Response: We respectfully thank the reviewer for this important methodological concern. We acknowledge that convenience sampling may limit external validity and population-level generalizability, and this limitation has already been explicitly recognized in the manuscript. However, the primary objective of the present study was exploratory and hypothesis-generating in nature, aimed at assessing associations between disease severity, psychological distress, and QoL among UC patients attending a tertiary care center in Pakistan, where such data remain limited.

The inferential statistical methods employed, including correlation analysis, group comparisons, and regression modeling, were used to evaluate internal associations within the studied cohort rather than to claim definitive population-level causation or national prevalence estimates. Similar analytical approaches have been widely used in observational hospital-based studies utilizing non-probability sampling, particularly in clinical and psychosocial research settings.

We have clarified that findings should be interpreted within the context of the sampled population and should not be generalized without confirmation from larger multicenter probability-based studies. Future prospective and multicenter investigations have also been recommended to validate and extend these findings. We appreciate your keen interest in reviewing the manuscript and hope that the mentioned clarification will be helpful in understanding the main theme.

On behalf of all authors, I would like to thank the reviewers for their valuable time reviewing this manuscript. If there are still any queries, we will happily respond and cooperate.

Kind Regards,

Research Team

Amjad Khan, PhD (Corresponding Author)

E-mail: amjadkhan@qau.edu.pk

Attachment Submitted filename: Response file 4.docx

# Peer review: Decision Letter 1

**DOI:** 10.1371/journal.pone.0356531.r003

**Authors:** Lubna Shirin

15 Jul 2026

Dear Dr. Khan,

Thank you for submitting your manuscript to PLOS ONE. After careful consideration, we feel that it has merit but does not fully meet PLOS ONE’s publication criteria as it currently stands. Therefore, we invite you to submit a revised version of the manuscript that addresses the points raised during the review process.

Please submit your revised manuscript by Aug 29 2026 11:59PM. If you will need more time than this to complete your revisions, please reply to this message or contact the journal office at plosone@plos.org. When you're ready to submit your revision, log on to https://www.editorialmanager.com/pone/ and select the 'Submissions Needing Revision' folder to locate your manuscript file.

- A letter that responds to each point raised by the academic editor and reviewer(s). You should upload this letter as a separate file labeled 'Response to Reviewers'.
- A marked-up copy of your manuscript that highlights changes made to the original version. You should upload this as a separate file labeled 'Revised Manuscript with Track Changes'.
- An unmarked version of your revised paper without tracked changes. You should upload this as a separate file labeled 'Manuscript'.

If you would like to make changes to your financial disclosure, please include your updated statement in your cover letter. Guidelines for resubmitting your figure files are available below the reviewer comments at the end of this letter.

If applicable, we recommend that you deposit your laboratory protocols in protocols.io to enhance the reproducibility of your results. Protocols.io assigns your protocol its own identifier (DOI) so that it can be cited independently in the future. For instructions see: https://journals.plos.org/plosone/s/submission-guidelines#loc-laboratory-protocols. Additionally, PLOS ONE offers an option for publishing peer-reviewed Lab Protocol articles, which describe protocols hosted on protocols.io. Read more information on sharing protocols at https://plos.org/protocols?utm_medium=editorial-email&utm_source=authorletters&utm_campaign=protocols.

As the corresponding author, your ORCID iD is verified in the submission system and will appear in the published article. PLOS supports the use of ORCID, and we encourage all coauthors to register for an ORCID iD and use it as well. Please encourage your coauthors to verify their ORCID iD within the submission system before final acceptance, as unverified ORCID iDs will not appear in the published article. *Only*  the individual author can complete the verification step; PLOS staff *cannot*  verify ORCID iDs on behalf of authors.

We look forward to receiving your revised manuscript.

Kind regards,

Lubna Shirin, Ph.D, M.Phil, MBBS

Academic Editor

PLOS One

Journal Requirements:

If the reviewer comments include a recommendation to cite specific previously published works, please review and evaluate these publications to determine whether they are relevant and should be cited. There is no requirement to cite these works unless the editor has indicated otherwise.

Additional Editor Comments:

Dear Dr. Amjad Khan,

Assalamualikum.

Kindly do your correction according to the reviewer comment and submit it again.

Kind Regards,

Dr. Lubna

[Note: HTML markup is below. Please do not edit.]

Reviewers' comments:

Reviewer's Responses to Questions

**Comments to the Author**

Reviewer #1: All comments have been addressed

Reviewer #3: (No Response)

**********

2. Is the manuscript technically sound, and do the data support the conclusions?

Reviewer #1: Yes

Reviewer #3: Partly

**********

3. Has the statistical analysis been performed appropriately and rigorously?

Reviewer #1: Yes

Reviewer #3: Yes

**********

4. Have the authors made all data underlying the findings in their manuscript fully available?

The PLOS Data policy

Reviewer #1: Yes

Reviewer #3: Yes

**********

5. Is the manuscript presented in an intelligible fashion and written in standard English?

Reviewer #1: Yes

Reviewer #3: No

**********

Reviewer #1: Thank you for addressing the comments raised during the previous review round. The revised manuscript has improved substantially, and most of the methodological and reporting concerns have been adequately addressed.

I have only one minor point for clarification. The reported mean values for HADS-A, HADS-D, and BDI-II appear to represent categorized scores rather than the original questionnaire scores. To avoid potential confusion for readers, please clarify this explicitly in the Methods and Results sections and indicate whether these values reflect category coding or raw scale scores.

With this minor clarification, I believe the manuscript is suitable for publication.

Reviewer #3: Dear Authors,

I. Title:

The term “impact” may imply a causal relationship between ulcerative colitis and the assessed outcomes. However, given the cross-sectional design of the study, causality cannot be established, as exposure and outcomes are assessed simultaneously. Therefore, from a methodological perspective, a title referring to an "association" or "relationship" rather than an "impact" would be more appropriate. For example: Mental Health and Quality of Life Among Patients with Ulcerative Colitis: A Cross-Sectional Study in a Pakistani Cohort or Association Between Ulcerative Colitis, Mental Health, and Quality of Life in a Pakistani Cohort or Assessment of Mental Health and Quality of Life in Patients with Ulcerative Colitis: A Cross-Sectional Analysis

II. Introduction:

Line:117-118 The sentence "In Pakistan, the prevalence of UC appears to be increasing (9), likely due to lifestyle changes, urbanization, dietary shifts, and better diagnostic capacities. " is somewhat misleading, as diagnostic capacity affects disease detection and the estimation of prevalence rather than necessarily contributing to a true increase in disease occurrence. In this case, it would be more appropriate to replace the sentence with the following: "In Pakistan, the prevalence of UC appears to be increasing (9), potentially as a result of lifestyle changes, urbanization, and dietary transitions. In addition, advances in diagnostic capabilities may have improved case detection, thereby enhancing the accuracy of prevalence estimates"

III. Methodology:

1. I understand that the study population consisted of Punjabi participants, as the authors state that the questionnaires were translated into Urdu. This information should be clearly specified in the Materials section.

2. Consider simplifying the description accompanying Table 1. to improve clarity and readability.

3. The authors should also specify the version of the Declaration of Helsinki referenced (e.g., the Declaration of Helsinki, 1975, revised in 2000).

4. Additionally, demographic information on the participants’ age is lacking and should be included.

5. The manuscript does not clearly indicate whether the patients with UC had any additional chronic diseases or comorbid conditions.

6. The manuscript lacks information on the therapies received by the patients enrolled in the study, which may be relevant for the interpretation of the results.

7. The methodology section requires linguistic revision and simplification to enhance readability and comprehension.

IV. Disscusion:

1. The first paragraph of the Discussion would benefit from revision and condensation. In addition, the core of the Discussion should provide a more in-depth discussion of the results in the context of the existing literature, particularly studies conducted by other authors addressing the same research topic, eg. doi: 10.1159/000538618; doi: 10.1159/000528788; doi: 10.1097/PG9.0000000000000287)

Major issue:

2. A major limitation that undermines the reliability of the conclusions is the small sample size, particularly given that all participants were recruited from a single center. This substantially limits the generalizability of the findings and does not allow for drawing conclusions applicable to the entire Pakistani population. The authors should acknowledge this limitation more explicitly and interpret their results with greater caution.

3. In addition, I suggest revising and shortening the first paragraph of the Discussion section. The main body of the Discussion should be expanded to provide a more comprehensive comparison of the findings with those reported by other authors investigating this topic.

Minor issue:

1) It is generally recommended to avoid starting sentences with abbreviations.

2) There are several inconsistencies in the formatting of the reference list that should be corrected to ensure compliance with the journal's guidelines.

3) A thorough language revision is recommended to improve clarity, readability, and grammatical accuracy.

4) The term “race” should preferably be replaced with “ethnic origin” (or “ethnicity”), as it is more appropriate and precise in this context.

Summary:

The study addresses an important and clinically relevant topic; however, several issues

should be addressed before the manuscript can be considered for publication. In particular,

the authors should provide additional information regarding patient characteristics

(including comorbidities, treatment regimens, and ethnic background), and strengthen the

Discussion section by placing their findings in a broader context of the existing literature.

Addressing these points would improve the clarity, scientific rigor, and overall impact of

the manuscript.

**********

what does this mean?). If published, this will include your full peer review and any attached files.

If you choose “no”, your identity will remain anonymous but your review may still be made public.

**Do you want your identity to be public for this peer review?**  For information about this choice, including consent withdrawal, please see our Privacy Policy

Reviewer #1: No

Reviewer #3: **Yes:** Grażyna Adler

**********

[NOTE: If reviewer comments were submitted as an attachment file, they will be attached to this email and accessible via the submission site. Please log into your account, locate the manuscript record, and check for the action link "View Attachments". If this link does not appear, there are no attachment files.]

To ensure your figures meet our technical requirements, please review our figure guidelines: https://journals.plos.org/plosone/s/figures

You may also use PLOS’s free figure tool, NAAS, to help you prepare publication quality figures: https://journals.plos.org/plosone/s/figures#loc-tools-for-figure-preparation.

NAAS will assess whether your figures meet our technical requirements by comparing each figure against our figure specifications.

# Author comment: Author response to Decision Letter 2

**DOI:** 10.1371/journal.pone.0356531.r004

22 Jul 2026

Author’s response to reviewers’ comments (Manuscript title: Association Between Ulcerative Colitis, Mental Health, and Quality of Life in a Pakistani Cohort: A Cross-Sectional Analysis)

Dear Editor,

Thank you for allowing us to submit a revised draft of our letter. I have attached a revised version of our manuscript, Association Between Ulcerative Colitis, Mental Health, and Quality of Life in a Pakistani Cohort: A Cross-Sectional Analysis, for evaluation and publication in PLOS One. We appreciate the time and effort you and the reviewers have dedicated to providing valuable feedback on our manuscript.

General Response

We are thankful to the reviewers for evaluating our manuscript. All the suggestions have been incorporated into the revised manuscript. The reviewers’ comments were constructive and included updated information. We hope these changes will clarify points and improve the overall presentation of our manuscript.

Here is a point-by-point response to the reviewers’ comments and concerns.

Reviewer 1

The reported mean values for HADS-A, HADS-D, and BDI-II appear to represent categorized scores rather than the original questionnaire scores. To avoid potential confusion for readers, please clarify this explicitly in the Methods and Results sections and indicate whether these values reflect category coding or raw scale scores.

With this minor clarification, I believe the manuscript is suitable for publication.

Response: We sincerely thank the reviewer for this constructive comment and for the positive evaluation of our manuscript. As suggested, we have revised the Methodology section (page 7, line 188) to clarify that the reported mean values for HADS-A, HADS-D, and BDI-II represent category-coded scores used in statistical analyses rather than the original cumulative raw questionnaire scores. We have also stated that no transformation or rescaling of the original questionnaire scores was performed, and that participant classification into normal, borderline, abnormal, or severity categories was based on the standard validated cut-off values for each instrument. We appreciate the reviewer's valuable suggestion, which has improved the manuscript's clarity and interpretability.

Reviewer 2

The term “impact” may imply a causal relationship between ulcerative colitis and the assessed outcomes. However, given the cross-sectional design of the study, causality cannot be established, as exposure and outcomes are assessed simultaneously. Therefore, from a methodological perspective, a title referring to an "association" or "relationship" rather than an "impact" would be more appropriate. For example: Mental Health and Quality of Life Among Patients with Ulcerative Colitis: A Cross-Sectional Study in a Pakistani Cohort or Association Between Ulcerative Colitis, Mental Health, and Quality of Life in a Pakistani Cohort or Assessment of Mental Health and Quality of Life in Patients with Ulcerative Colitis: A Cross-Sectional Analysis

Response: We sincerely thank the reviewer for this valuable suggestion. The manuscript title has been revised and updated in accordance with your recommendation to more accurately reflect the scope, objectives, and key findings of the study. We appreciate your constructive feedback, which has improved the title's clarity and relevance.

Line:117-118 The sentence "In Pakistan, the prevalence of UC appears to be increasing (9), likely due to lifestyle changes, urbanization, dietary shifts, and better diagnostic capacities. " is somewhat misleading, as diagnostic capacity affects disease detection and the estimation of prevalence rather than necessarily contributing to a true increase in disease occurrence. In this case, it would be more appropriate to replace the sentence with the following: "In Pakistan, the prevalence of UC appears to be increasing (9), potentially as a result of lifestyle changes, urbanization, and dietary transitions. In addition, advances in diagnostic capabilities may have improved case detection, thereby enhancing the accuracy of prevalence estimates"

Response: We thank the reviewer for the careful evaluation of our manuscript. The suggested revision has been addressed, and the manuscript has been comprehensively updated.

Methodology:

1. I understand that the study population consisted of Punjabi participants, as the authors state that the questionnaires were translated into Urdu. This information should be clearly specified in the Materials section.

Response: We thank the reviewer for this observation. We would like to clarify that the study population consisted of Pakistani patients, not exclusively Punjabi participants. Urdu is the national language of Pakistan and is widely understood across all provinces, irrespective of participants' native languages. Therefore, the validated Urdu versions of the questionnaires were used to ensure standardized administration and facilitate comprehension among all participants.

2. Consider simplifying the description accompanying Table 1. to improve clarity and readability.

Response: We thank the reviewer for this recommendation. The description accompanying Table 1 presents only the individual components of the Mayo score used to assess disease activity, rather than the baseline clinical characteristics or presenting symptoms of the study participants (page 6, lines 146-153).

3. The authors should also specify the version of the Declaration of Helsinki referenced (e.g., the Declaration of Helsinki, 1975, revised in 2000).

Response: We thank the reviewer for this helpful suggestion. The Ethics Statement has been updated to specify the version of the Declaration of Helsinki followed in this study (page 5, line 142).

4. Additionally, demographic information on the participants’ age is lacking and should be included.

Response: We thank the reviewer for this comment. We would like to clarify that the demographic information on participants' ages has already been reported in the first paragraph of the Results section of the manuscript (Page 8, Line 207).

5. The manuscript does not clearly indicate whether the patients with UC had any additional chronic diseases or comorbid conditions.

Response: We thank the reviewer for this important observation. We would like to clarify that patients with additional chronic diseases or significant comorbidities were excluded per the study eligibility criteria to minimize potential confounding. This has now been explicitly stated in the methodology to improve clarity regarding the study population.

6. The manuscript lacks information on the therapies received by the patients enrolled in the study, which may be relevant for the interpretation of the results.

Response: We thank the reviewer for this important comment. We acknowledge that treatment may influence psychological well-being and QoL in patients with UC. However, the primary objective of the present study was to evaluate the association between disease severity and psychological outcomes, rather than treatment effectiveness. Since patients were receiving heterogeneous treatment regimens based on their individual clinical status, disease severity, and the treating physician's judgment, meaningful comparison of therapeutic effects was beyond the scope of this study and could have introduced substantial heterogeneity. Therefore, treatment-related analyses were not performed. We have clarified this point and acknowledged it as a limitation, and we recommend that future prospective studies investigate the impact of specific therapeutic regimens on psychological distress and QoL in UC patients.

7. The methodology section requires linguistic revision and simplification to enhance readability and comprehension.

Response: We are grateful to the reviewer for the insightful comments and recommendations. The manuscript has been revised accordingly, and all relevant sections have been updated to address the concerns raised. We appreciate the valuable feedback, which has strengthened the overall quality of the manuscript.

Discussion.

1. The first paragraph of the Discussion would benefit from revision and condensation. In addition, the core of the Discussion should provide a more in-depth discussion of the results in the context of the existing literature, particularly studies conducted by other authors addressing the same research topic, eg. doi: 10.1159/000538618; doi: 10.1159/000528788; doi: 10.1097/PG9.0000000000000287)

Response: We sincerely thank the reviewer for this constructive suggestion. In response, we have substantially revised the Discussion section by condensing the introductory paragraph to improve focus and readability.

Major issue:

2. A major limitation that undermines the reliability of the conclusions is the small sample size, particularly given that all participants were recruited from a single center. This substantially limits the generalizability of the findings and does not allow for drawing conclusions applicable to the entire Pakistani population. The authors should acknowledge this limitation more explicitly and interpret their results with greater caution.

Response: We thank the reviewer for this important observation. The limitation regarding the relatively small sample size has now been explicitly acknowledged in the limitations section (page 12, line 351). We have clarified that the modest sample size, together with the single-center design, may limit the generalizability of the findings. This has also been highlighted as an area for future research, with recommendations for larger, multicenter studies to validate and extend the present findings.

3. In addition, I suggest revising and shortening the first paragraph of the Discussion section. The main body of the Discussion should be expanded to provide a more comprehensive comparison of the findings with those reported by other authors investigating this topic.

Response: We sincerely thank the reviewer for this valuable suggestion. The Discussion section has been comprehensively revised and updated to improve its structure, reduce repetition, and provide a more focused interpretation of the findings. Additional comparisons with relevant published literature have been incorporated to better contextualize the results, thereby strengthening the scientific discussion and the manuscript's overall quality.

Minor issue:

1) It is generally recommended to avoid starting sentences with abbreviations.

Response: We thank the reviewer for this helpful suggestion. The manuscript has been revised accordingly, and sentences beginning with abbreviations have been rephrased to improve readability and conform to standard scientific writing conventions.

2) There are several inconsistencies in the formatting of the reference list that should be corrected to ensure compliance with the journal's guidelines.

Response: We thank the reviewer for this valuable observation. The reference list has been carefully reviewed and updated using reference management software to ensure consistent formatting, accurate citations, and compliance with the journal's referencing style. All in-text citations and corresponding references have been cross-checked and harmonized throughout the manuscript.

3) A thorough language revision is recommended to improve clarity, readability, and grammatical accuracy.

Response: We thank the reviewer for the careful evaluation of our manuscript. The suggested revisions have been addressed, and the manuscript has been comprehensively updated.

4) The term “race” should preferably be replaced with “ethnic origin” (or “ethnicity”), as it is more appropriate and precise in this context.

Response: We are grateful to the reviewer for the insightful recommendations, and we have updated all relevant sections to address the concerns raised.

On behalf of all authors, I would like to thank the reviewers for their valuable time reviewing this manuscript. If there are still any queries, we will happily respond and cooperate.

Kind Regards,

Research Team

Amjad Khan, PhD (Corresponding Author)

E-mail: amjadkhan@qau.edu.pk

Attachment Submitted filename: Response file 5.docx

# Editor report: Decision Letter 2

**DOI:** 10.1371/journal.pone.0356531.r005

**Authors:** Lubna Shirin

4 Aug 2026

Association Between Ulcerative Colitis, Mental Health, and Quality of Life in a Pakistani Cohort: A Cross-Sectional Analysis

PONE-D-26-05459R2

Dear Dr. Amjad,

We’re pleased to inform you that your manuscript has been judged scientifically suitable for publication and will be formally accepted for publication once it meets all outstanding technical requirements.

Within one week, you’ll receive an e-mail detailing the required amendments. When these have been addressed, you’ll receive a formal acceptance letter and your manuscript will be scheduled for publication.

An invoice will be generated when your article is formally accepted. Please note, if your institution has a publishing partnership with PLOS and your article meets the relevant criteria, all or part of your publication costs will be covered. Please make sure your user information is up-to-date by logging into Editorial Manager at Editorial Manager® and clicking the ‘Update My Information' link at the top of the page. For questions related to billing, please contact billing support.

If your institution or institutions have a press office, please notify them about your upcoming paper to help maximize its impact. If they’ll be preparing press materials, please inform our press team as soon as possible -- no later than 48 hours after receiving the formal acceptance. Your manuscript will remain under strict press embargo until 2 pm Eastern Time on the date of publication. For more information, please contact onepress@plos.org.

Kind regards,

Lubna Shirin, Ph.D, M.Phil, MBBS

Academic Editor

PLOS One

Additional Editor Comments (optional):

Reviewers' comments:

# Editor report: Acceptance letter

**DOI:** 10.1371/journal.pone.0356531.r006

**Authors:** Lubna Shirin

PONE-D-26-05459R2

PLOS One

Dear Dr. Khan,

I'm pleased to inform you that your manuscript has been deemed suitable for publication in PLOS One. Congratulations! Your manuscript is now being handed over to our production team.

At this stage, our production department will prepare your paper for publication. This includes ensuring the following:

* All references, tables, and figures are properly cited

* All relevant supporting information is included in the manuscript submission,

* There are no issues that prevent the paper from being properly typeset

You will receive further instructions from the production team, including instructions on how to review your proof when it is ready. Please keep in mind that we are working through a large volume of accepted articles, so please give us a few days to review your paper and let you know the next and final steps.

Lastly, if your institution or institutions have a press office, please let them know about your upcoming paper now to help maximize its impact. If they'll be preparing press materials, please inform our press team within the next 48 hours. Your manuscript will remain under strict press embargo until 2 pm Eastern Time on the date of publication. For more information, please contact onepress@plos.org.

You will receive an invoice from PLOS for your publication fee after your manuscript has reached the completed accept phase. If you receive an email requesting payment before acceptance or for any other service, this may be a phishing scheme. Learn how to identify phishing emails and protect your accounts at https://explore.plos.org/phishing.

If we can help with anything else, please email us at customercare@plos.org.

Thank you for submitting your work to PLOS One and supporting open access.

Kind regards,

PLOS ONE Editorial Office Staff

on behalf of

Dr. Lubna Shirin

Academic Editor

PLOS One
